# NUP85 Mediates Endoplasmic Reticulum Stress through the USP47/ASK1 Signaling Pathway to Regulate the Progression of Liver Fibrosis

**DOI:** 10.1002/advs.202519972

**Published:** 2026-03-28

**Authors:** Dashuai Yang, Haoran Yang, Linxin Pan, Chang Tian, Fucheng Zuo, Shilei Huang, Xianrui Li, Ming Chen, Cheng Qian, Jie Wang, Zhaolin Chen, Tao Xu

**Affiliations:** ^1^ Inflammation and Immune Mediated Diseases Laboratory of Anhui Province School of Pharmacy Anhui Medical University Hefei China; ^2^ Anhui Institute of Medicine Hefei China; ^3^ School of Life Science Anhui Medical University Hefei China; ^4^ Center for Scientific Research Auhui Medical University Hefei China; ^5^ Department of Emergency Surgery The Second Affiliated Hospital of Anhui Medical University Hefei China; ^6^ Department of Pharmacy The First Affiliated Hospital of USTC Division of Life Sciences and Medicine University of Science and Technology of China Hefei Anhui China; ^7^ Anhui Provincial Key Laboratory of Precision Pharmaceutical Preparations and Clinical Pharmacy Hefei Anhui China

**Keywords:** ASK1, endoplasmic reticulum stress, liver fibrosis, NUP85, USP47

## Abstract

Liver fibrosis is a pathological process caused by excessive deposition of extracellular matrix (ECM) in the liver stimulated by chronic injury or inflammation. Nuclear pore protein 85 (NUP85) has been implicated in the development of various liver diseases. However, its involvement in liver fibrosis remains unclear. The present study aimed to explore the role of NUP85 in liver fibrosis. The expression level of NUP85 was found to be elevated in the liver tissues of liver fibrosis patients and mice. Knockdown of NUP85 not only ameliorated liver injury and collagen deposition, but also suppressed endoplasmic reticulum stress (ERS). Conversely, the opposite pathological and biochemical changes are observed with NUP85 overexpression. Mechanistically, NUP85 competitively binds ubiquitin‐specific peptidase 47 (USP47) to apoptosis signal‐regulating kinase 1 (ASK1), deubiquitinates lysine residue 805 of ASK1, and regulates the activation of ASK1, thereby exacerbating collagen deposition and ERS. Furthermore, we developed a CREKA‐coupled liposome as a targeted delivery system to deliver Mogroside V (MV), a pharmacological inhibitor of NUP85, to activated HSCs and attenuate liver fibrosis. Taken together, the present study demonstrated that NUP85 is a novel regulator of liver fibrosis and that the NUP85‐USP47‐ASK1 signaling pathway might be a strategy for therapeutic intervention.

## Graphical abstract

In the present study, we have demonstrated that the NUP85‐USP47‐ASK1 signaling pathway may have regulated the progression of liver fibrosis through modulating ERS. Additionally, we have developed a CREKA‐coupled liposome to target delivery of MV, a pharmacological inhibitor of NUP85, to activated HSCs, thereby attenuating liver fibrosis.

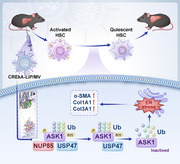

## 1 Introduction

Liver fibrosis is the pathological repair response of the liver to chronic injury and is a critical step in the progression of various chronic liver diseases to cirrhosis [1, 2]. The updated data show that pooled prevalence rates of advanced liver fibrosis in the general population were 3.3% (95% CI, 2.4%–4.2%) worldwide [3]. Liver fibrosis is characterized by excessive accumulation of extracellular matrix (ECM), which can further deteriorate into cirrhosis or primary hepatocellular carcinoma (HCC) if left untreated [4, 5]. Unlike cirrhosis and HCC, liver fibrosis remains potentially reversible when lobular architecture remains intact and functional reserve is preserved [6, 7]. This pathophysiological feature provides a critical therapeutic window to prevent fibrosis progression prior to irreversible liver decompensation. Current clinical strategies prioritize etiological management due to the lack of clinically approved anti‐fibrotic agents that can effectively halt or reverse ECM remodeling [8, 9]. Therefore, it is crucial to explore the mechanisms underlying liver fibrosis and to identify new therapeutic methods for this condition.

Nuclear pore protein 85 (NUP85) is an important component of the nuclear pore complex and is involved in nucleoplasmic transport and the exchange of substances within and outside the nucleus [10, 11, 12]. Previous studies have shown that NUP85 is involved in the development of several liver diseases, including non‐alcoholic fatty liver disease (NAFLD) and HCC [11, 13]. In NAFLD, impaired chaperone‐mediated autophagy‐induced NUP85 degradation exacerbates monocyte recruitment, which promotes liver inflammation and disease progression in NAFLD [12]. Additionally, the high expression level of NUP85 in HCC is correlated with a poor prognosis and is related to various immune cells and drugs, making it a potential biomarker for diagnosis, treatment, and prognosis in HCC [13]. Notably, recent findings from our group suggested that NUP85 regulated the PI3K/AKT signaling pathway through interaction with C‐C motif chemokine receptor 2 (CCR2), thereby affecting lipid homeostasis and inflammation in NAFLD [11]. Additionally, there were findings suggested that liver fibrosis could be ameliorated by inhibiting CCR2 to restore the immune cell landscape. However, the role of NUP85 in liver fibrosis is unclear.

In the present study, NUP85 was significantly upregulated in liver fibrosis, and that knockdown of NUP85 attenuated the activation of endoplasmic reticulum stress (ERS) in vivo and in vitro, thereby inhibiting the activation of HSCs. Mechanistically, NUP85 deubiquitinates lysine residue 805 of apoptosis signal‐regulating kinase 1 (ASK1) via ubiquitin‐specific peptidase 47 (USP47) and regulates ASK1 activation. Notably, reducing the expression level of NUP85 with Cys‐Arg‐Glu‐Lys‐Ala‐modified liposomes/Mogroside V (CREKA‐Lip/MV) attenuated liver fibrosis in CCl_4_‐induced mice. Collectively, these data reveal the essential role of NUP85 in liver fibrosis and highlight the potential of targeting NUP85 as a therapeutic for liver fibrosis.

## 2 Results

### 2.1 NUP85 Expression Level is Elevated in Liver Fibrosis

**FIGURE 1:**
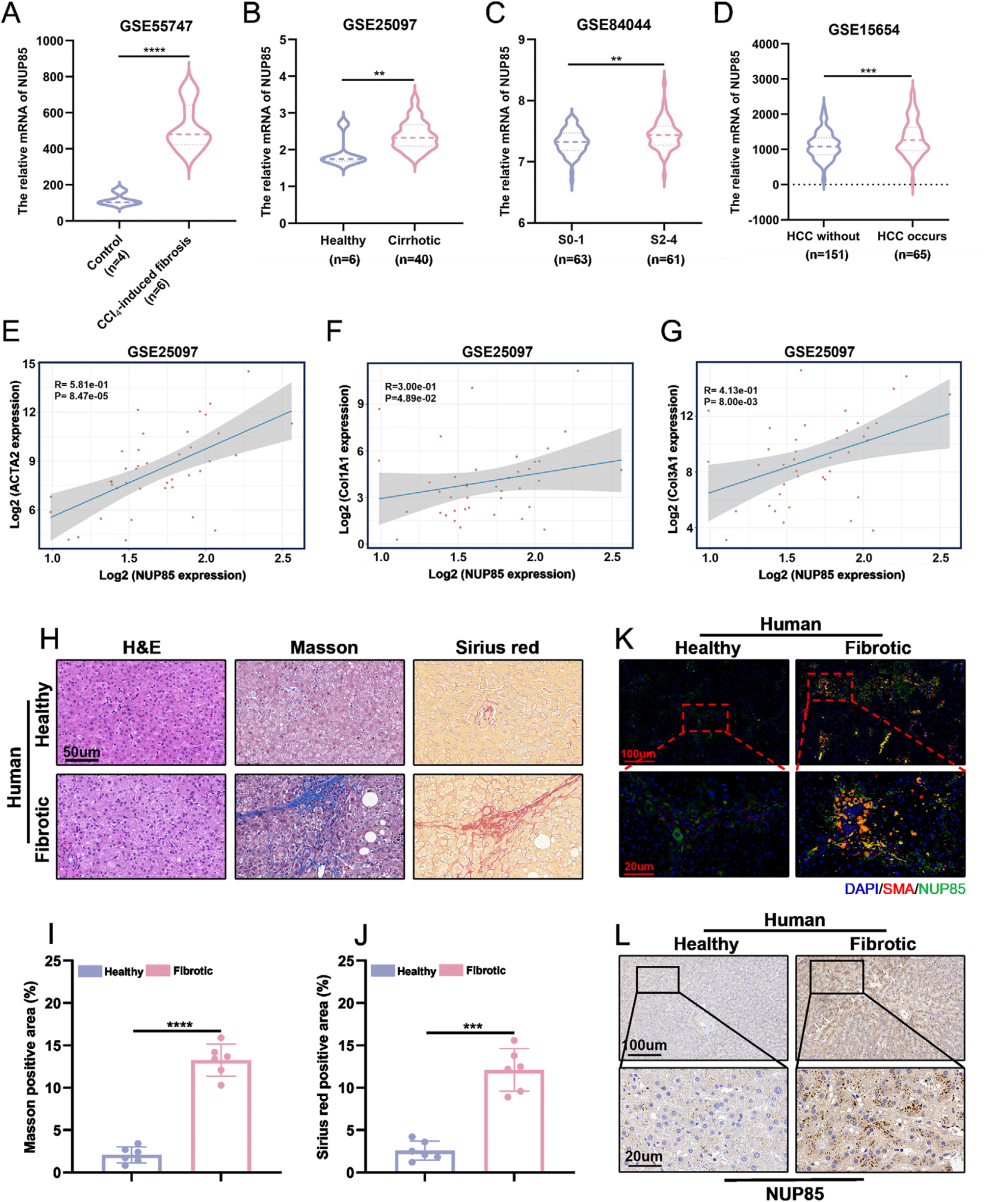
NUP85 expression level was elevated in liver fibrosis. (A) NUP85 expression level in CCl_4_‐induced liver fibrosis and normal control from GSE55747. (B) NUP85 expression level in liver cirrhosis and normal control from GSE25097. (C) NUP85 expression level in patients with Scheuer scores S0‐1 and S2‐4 from GSE84044. (D) NUP85 expression level in cirrhotic patients who develop HCC and those who do not develop HCC from GSE15654. (E–G) Correlation analysis of NUP85 expression level with ACTA2, Col1A1, and Col3A1 expression levels in cirrhotic samples from GSE25097. (H–J) Representative H&E staining, Masson staining, and Sirius red staining images, Masson positive area (%), and Sirius red positive area (%) in liver tissues of mice (scale bars, 50 µm. n = 6). (K, L) NUP85 expression level in liver tissues of human patients with liver fibrosis was detected by IF and IHC (scale bars, 100, 20 µm. n = 6). All data are presented as the mean ± SD (n  = 3 independent experiments). Levels of statistical significance are indicated as ^**^ *p* < 0.01, ^***^ *p* < 0.001, ^****^ *p* < 0.0001. One‐way ANOVA with Tukey test analysis and a two‐tailed Student *t* test were used for statistical analysis.

To evaluate the correlation between the expression level of NUP85 and the progression of liver fibrosis, the expression level of NUP85 was evaluated through examining multiple Gene Expression Omnibus (GEO) datasets. Bioinformatic analysis result of the GSE55747 dataset revealed a significant upregulation of NUP85 expression level in CCl_4_‐induced liver fibrosis (Figure 1A). In concurrence with the finding from the GSE25097 dataset, NUP85 expression level was significantly upregulated in cirrhotic liver tissues compared to healthy liver tissues (Figure 1B). Moreover, the analysis result of the GSE84044 dataset showed that NUP85 expression level was upregulated in patients with advanced fibrosis (Scheuer stages S2‐4) compared to those with mild or no fibrosis (S0‐1) (Figure 1C). Further analysis result of the cirrhosis dataset (GSE15654) indicated that hepatocellular carcinoma (HCC) patients had significantly upregulated NUP85 expression level compared to non‐HCC cirrhosis patients (Figure 1D). In cirrhotic samples from the GSE25097 dataset (n = 40), positive correlations were observed between expression level of NUP85 and fibrosis markers: ACTA2 (p = 8.47e‐05, r = 0.581), Col1A1 (p = 4.89e‐02, r = 0.300), and Col3A1 (p = 8.00e‐03, r = 0.413) (Figure 1E–G).

To further evaluate the potential utility of NUP85 as a marker for liver fibrosis, human liver tissue samples were obtained, including normal and cirrhotic samples (n = 6 per group). The results of H&E staining, Masson staining, and Sirius Red staining showed that the liver parenchyma of cirrhotic tissues were structurally disturbed with characteristic fibrosis changes (Figure 1H–J). Additionally, the expression level of NUP85 was found to be increased in human liver fibrosis samples by immunofluorescence (IF), immunohistochemistry (IHC), Western blotting and RT‐qPCR (Figure 1K,L; Figure S1A,B). Similarly, Western blotting, RT‐qPCR, IF, and IHC results showed that the expression level of NUP85 was elevated in the liver tissues of liver fibrosis mice (Figure S1C–G). In addition, the expression level of NUP85 was elevated in LX‐2 cells (Human hepatic stellate cell line) activated by TGF‐β1 stimulation compared to non‐activated LX‐2 cells (Figure S1H–K). Next, to investigate changes of NUP85 expression level in primary cells, mice received twice‐weekly intraperitoneal injections of CCl_4_ for 8 weeks, followed by isolation of primary hepatocytes, Kupffer cells, and HSCs (Figure S2A). Western blotting and RT‐qPCR results demonstrated that NUP85 expression was selectively upregulated in primary HSCs (Figure S2B–J). A similar phenomenon was observed in another liver fibrosis model, where mice underwent bile duct ligation (BDL) surgery for 2 weeks (Figure S2K–S). These results provide substantial evidence for a significant correlation between NUP85 expression level and the progression of liver fibrosis.

### 2.2 Knockdown of NUP85 Alleviates Liver Fibrosis in Mice

To investigate the role of NUP85 in the development of liver fibrosis, liver‐specific NUP85 knockdown mice was constructed. Mice were administered AAV8‐shRNA‐NUP85 or AAV8‐NC via tail vein injection with 8‐week CCl_4_ treatment (Figure S3A). AAV8‐shRNA‐NUP85 effectively reduced liver NUP85 expression level (Figure S4A–E). Serological analysis results showed that the expression levels of ALT, AST, and ALP were elevated in CCl_4_‐ induced mice. In addition, the expression levels of ALT, AST, and ALP were significantly decreased in CCl_4_‐induced liver‐specific NUP85 knockdown mice (Figure S3B–D). H&E staining result indicated that liver‐specific NUP85 knockdown alleviated liver injury in CCl_4_‐induced mice (Figure S3F). Masson staining, Sirius Red staining, and hydroxyproline quantification results indicated that liver‐specific NUP85 knockdown reduced collagen deposition in CCl_4_‐induced mice (Figure S3E–H). Similarly, the results of Western blotting, RT‐qPCR, and IHC showed that the expression levels of fibrosis markers (α‐SMA, Col1A1, and Col3A1) were significantly decreased in the livers of liver‐specific NUP85 knockdown mice (Figure S3F,I,J; Figure S4F,G). Moreover, similar conclusions were obtained through the BDL‐induced mice model (Figure S5). These data indicated that liver‐specific knockdown of NUP85 ameliorates liver fibrosis in mice.

**FIGURE 2:**
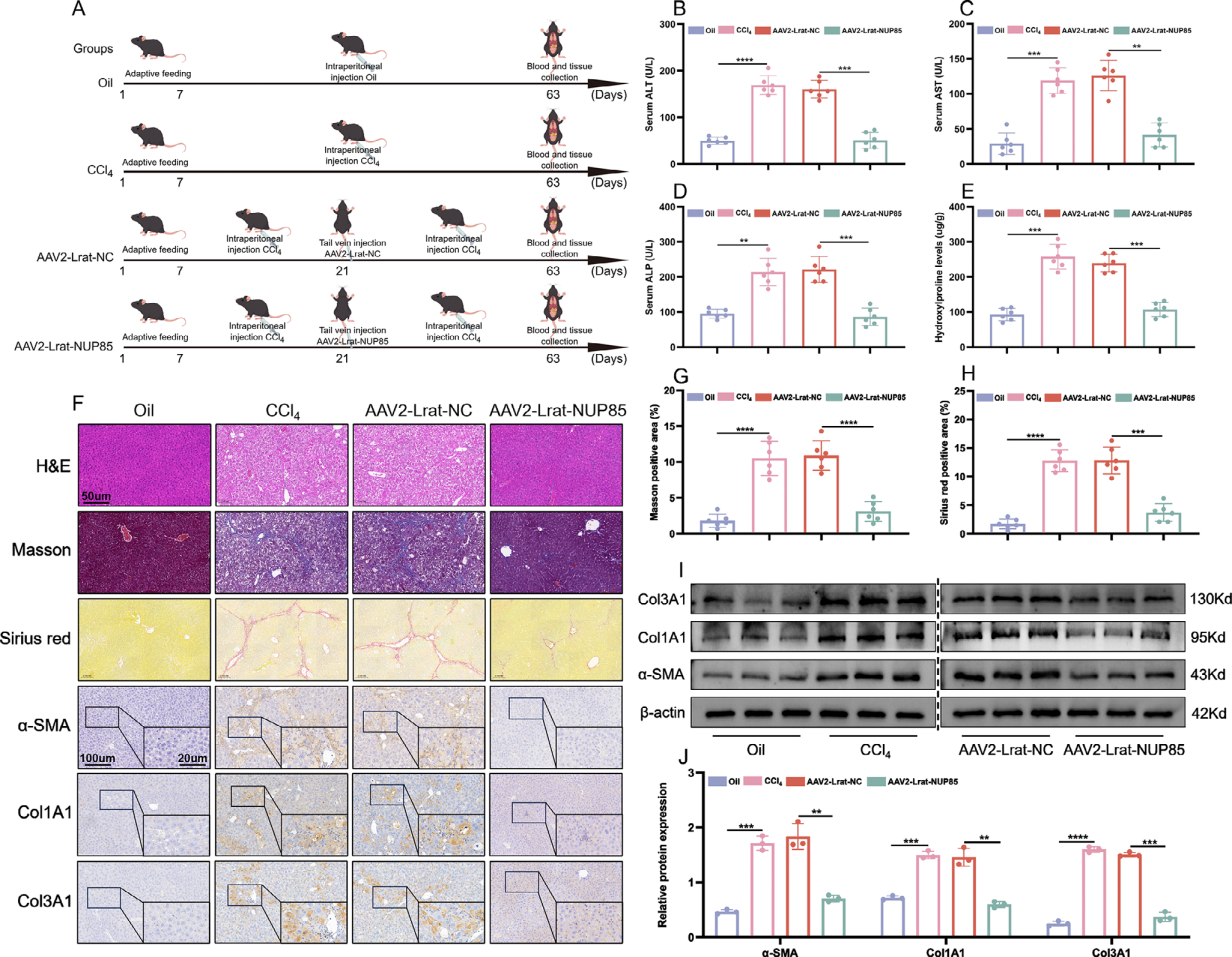
HSCs‐specific NUP85 knockdown alleviates CCl_4_‐induced liver fibrosis. (A) Schematic illustrating of the experimental design of AAV2‐Lrat‐NUP85 for the treatment of CCl_4_‐induced liver fibrosis in mice (n=6). (B–D) Serum expression levels of ALT, AST, and ALP in mice (n=6). (E) Liver hydroxyproline content in mice (n=6). (F–H) Representative H&E staining, Masson staining, Sirius red staining, and IHC images, Masson positive area (%), and Sirius red positive area (%) in liver tissues of mice (scale bars, 50, 100, 20 µm n=6). (I, J) Expression levels of α‐SMA, Col1A1, and Col3A1 in liver tissues of mice were detected by Western blotting and bar plot. All data are presented as the mean ± SD (n =3 independent experiments). Levels of statistical significance are indicated as ^**^ *p* < 0.01, ^***^ *p* < 0.001, ^****^ *p* < 0.0001. One‐way ANOVA with Tukey test analysis and a two‐tailed Student *t* test were used for statistical analysis.

To further investigate the role of NUP85 in HSCs, HSCs‐specific NUP85 knockdown mice were constructed. Mice were administered AAV2‐Lrat‐NC or AAV2‐Lrat‐NUP85 via tail vein injection with 8‐week CCl_4_ treatment (Figure 2A). Western blotting and RT‐qPCR results showed that the expression level of NUP85 in HSCs was significantly reduced by AAV2‐Lrat‐NUP85, while in hepatocytes and Kuffer cells were not affected (Figure S4I–Q). Serological analysis results showed that the expression levels of ALT, AST, and ALP were significantly reduced in AAV2‐Lrat‐NUP85‐treated CCl_4_‐induced mice compared to AAV2‐Lrat‐NC‐treated CCl_4_‐induced mice (Figure 2B–D). H&E staining result indicated that HSCs‐specific NUP85 knockdown alleviated liver injury in CCl_4_‐induced mice (Figure 2F). Masson staining, Sirius Red staining, and hydroxyproline quantification results indicated that HSCs‐specific NUP85 knockdown reduced collagen deposition in CCl_4_‐induced mice (Figure 2E–H). Similarly, the results of Western blotting, RT‐qPCR, and IHC showed that the expression levels of fibrosis markers (α‐SMA, Col1A1 and Col3A1) were significantly decreased in the livers of mice with HSCs‐specific knockdown of NUP85 (Figure 2F,I,J; Figure S4H). Moreover, similar conclusions were obtained through the BDL‐induced mice model (Figure S6). These data indicated that HSCs‐specific NUP85 knockdown ameliorates liver fibrosis in mice.

To further elucidate the role of NUP85 in the progression of liver fibrosis, NUP85 expression level were restored in NUP85 knockout mice (Figure S7A). Serological analysis revealed that restoring NUP85 expression significantly elevated serum levels of ALT, AST, and ALP in mice (Figure S7B–D). H&E staining revealed that restoring NUP85 expression exacerbated liver injury in CCl_4_‐induced mice (Figure S7F). Masson staining, Sirius Red staining, and hydroxyproline quantification results demonstrated that restoring NUP85 expression increased collagen deposition in CCl_4_‐induced mice (Figure S7E–H). Furthermore, Western blotting and IHC revealed markedly elevated expression of fibrosis markers (α‐SMA, Col1A1, and Col3A1) in the livers of AAV2‐Lrat‐oeNUP85 mice (Figure S7F,I,J). Moreover, similar conclusions were obtained through BDL‐induced mice model (Figure S8). These data indicated that HSC‐specific overexpression of NUP85 exacerbates liver fibrosis in mice.

### 2.3 Knockdown of NUP85 Inhibits LX‐2 Cells Activation

To investigate the effect of TGF‐β1 stimulation on the expression level of NUP85, LX‐2 cells were treated with different concentrations and times of TGF‐β1. Western blotting results revealed maximal NUP85 protein expression level occurred at 24 h post‐treatment with 10 ng/mL TGF‐β1 (Figure S9A–D). Subsequently, the transfection efficiency of NUP85‐siRNA and pcDNA3.1‐3‐NUP85 plasmid was validated. Western blotting, RT‐qPCR, and IF results demonstrated that the expression level of NUP85 was significantly reduced by transfecting NUP85‐siRNA in TGF‐β1‐induced LX‐2 cells. Similarly, the expression level of NUP85 was significantly elevated by transfecting pcDNA3.1‐3‐NUP85 in TGF‐β1‐induced LX‐2 cells (Figure S9E–L). Based on these results, the effect of NUP85 on the activation of TGF‐β1‐induced LX‐2 cells was further investigated.

**FIGURE 3:**
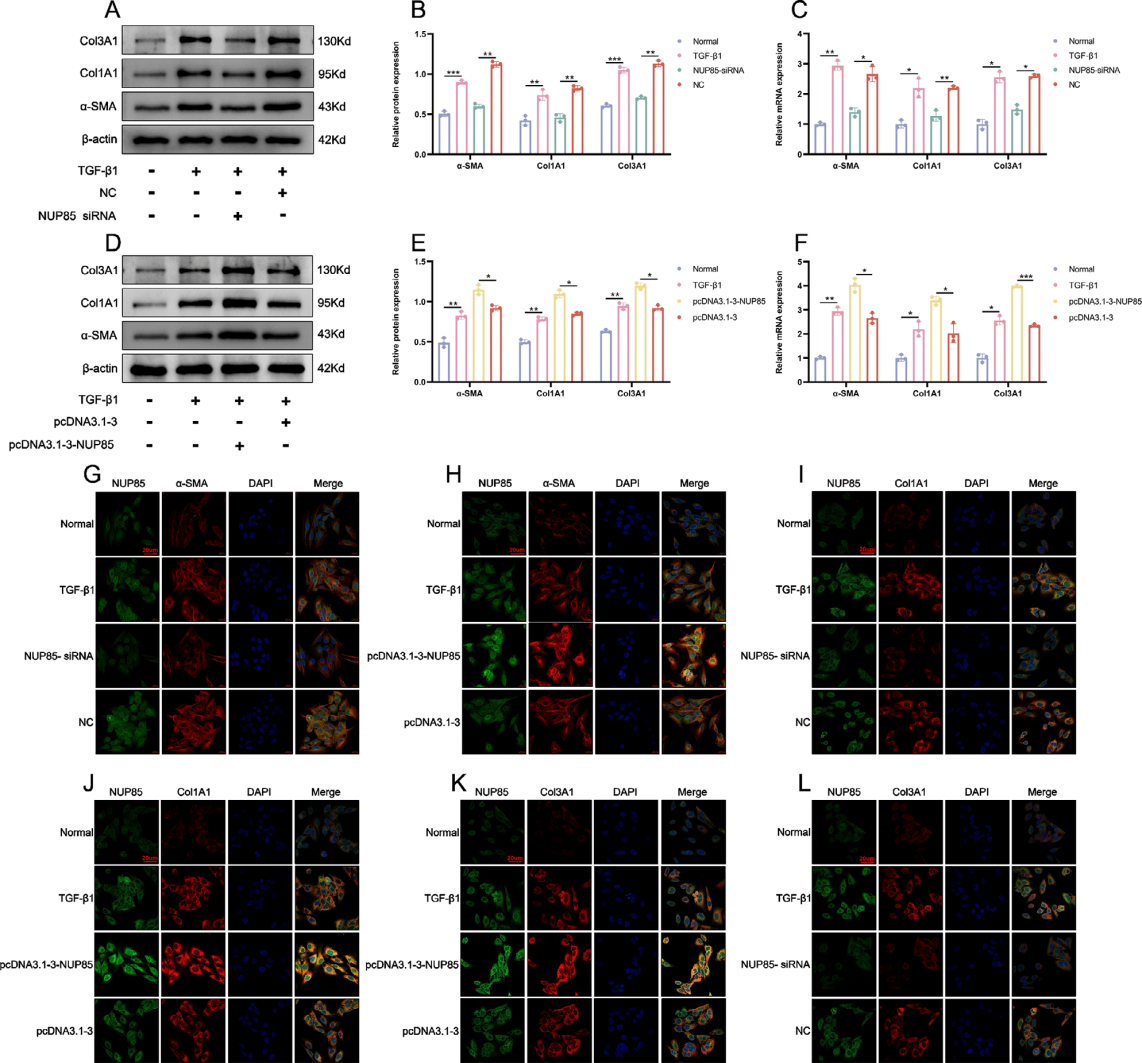
NUP85 knockdown inhibits TGF‐β1‐induced activation of HSCs. (A–F) Expression levels of α‐SMA, Col1A1, and Col3A1 in LX‐2 cells were detected by Western blotting and RT‐qPCR. (G–L) Expression levels of NUP85, α‐SMA, Col1A1, and Col3A1 in LX‐2 cells were detected by IF (scale bars, 20 µm). All data are presented as the mean ± SD (n =3 independent experiments). Levels of statistical significance are indicated as ^*^ *p* < 0.05, ^**^ *p* < 0.01, ^***^ *p* < 0.001. One‐way ANOVA with Tukey test analysis and a two‐tailed Student *t* test were used for statistical analysis.

Following validation of NUP85‐siRNA and pcDNA3.1‐3‐NUP85 transfection efficiency, the regulatory role of NUP85 on the activation of TGF‐β1‐induced LX‐2 cells was investigated. Western blotting, RT‐qPCR, and IF results demonstrated NUP85 knockdown significantly reduced α‐SMA, Col1A1, and Col3A1 expression levels in TGF‐β1‐induced LX‐2 cells (Figure 3A–C,G,I,L). Next, the effect of NUP85 overexpression on the activation of TGF‐β1‐induced LX‐2 cells was investigated. NUP85 overexpression consistently upregulated α‐SMA, Col1A1, and Col3A1 expression levels in TGF‐β1‐induced LX‐2 cells (Figure 3D–F,H,J,K). Finally, to investigate the effect of NUP85 on the spontaneous activation of primary HSCs in vitro, primary HSCs from mice were extracted. Western blotting, RT‐qPCR, and IF results showed elevated expression levels of α‐SMA, Col1A1, and Col3A1 in activated primary HSCs (Figure S10A,D,G,J). However, knockdown of NUP85 inhibited the activation of primary HSCs, as evidenced by reduced expression levels of α‐SMA, Col1A1, and Col3A1 (Figure S10B,C,E,F,H,I,K,L). In summary, these results suggested that knockdown of NUP85 inhibited TGF‐β1‐induced HSCs activation.

### 2.4 Knockdown of NUP85 Inhibited ERS in HSCs

**FIGURE 4:**
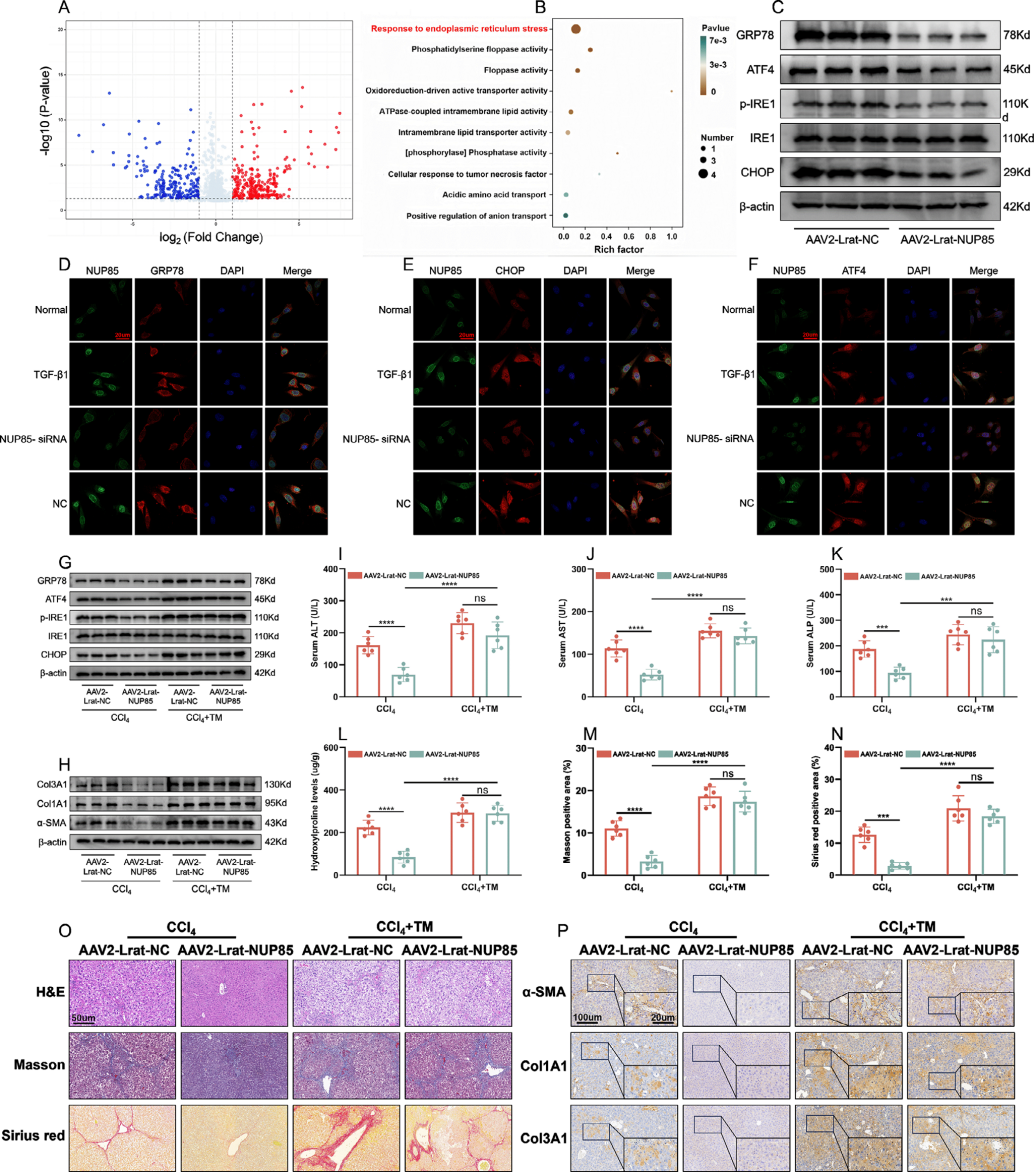
NUP85 knockdown alleviates CCl_4_‐induced liver fibrosis and TGF‐β1‐induced activation of HSCs through ERS. (A) Volcano plot representation of significantly up and downregulated genes. (B) GO analysis of significantly changed genes in biological processes. (C) Expression levels of GRP78, ATF4, p‐IRE1, IPE1, and CHOP in liver tissues of mice were detected by Western blotting. (D–F) Expression levels of NUP85, GRP78, ATF4, and CHOP in LX‐2 cells were detected by IF (scale bars, 20 µm). (G, H) Expression levels of GRP78, ATF4, p‐IRE1, IPE1, CHOP, α‐SMA, Col1A1, and Col3A1 in liver tissues of mice were detected by Western blotting. (I–K) Serum expression levels of ALT, AST, and ALP in mice (n=6). (L) Liver hydroxyproline content in mice (n=6). (M–P) Representative H&E staining, Masson staining, Sirius red staining, and IHC images, Masson positive area (%) and Sirius red positive area (%) in liver tissues of mice (scale bars, 50, 100, 20 µm. n=6). All data are presented as the mean ± SD (n =3 independent experiments). Levels of statistical significance are indicated as ^***^ *p* < 0.001, ^****^ *p* < 0.0001, “ns” indicates no significance. One‐way ANOVA with Tukey test analysis and a two‐tailed Student *t* test were used for statistical analysis.

To elucidate the molecular mechanisms underlying NUP85‐regulated liver fibrosis, RNA sequencing was conducted in liver tissue specimens from control and NUP85‐knockdown mice. Using p<0.05 and 1.5Xfold change as a cut‐off, 1309 differentially expressed genes (DEGs) were identified (Figure 4A). GO enrichment analysis result showed that knockdown of NUP85 affected ERS in liver fibrosis (Figure 4B). ERS is a common pathological response in liver fibrosis, and previous studies have shown that ERS promotes HSCs activation and exacerbates liver fibrosis through the activation of signaling pathways such as ATF6, IRE1α, and PERK [14, 15]. Thus, NUP85 regulation of liver fibrosis progression was speculated to be dependent on ERS. Additionally, Western blotting results confirmed that the expression levels of ERS markers (GRP78, ATF4, p‐IRE1, and CHOP) was increased in the liver tissues of CCl_4_‐induced mice, whereas the expression levels of these proteins were significantly reduced in the liver tissues of CCl_4_‐induced NUP85 knockdown mice (Figure 4C; Figure S11A). Similarly, Western blotting and IF results showed that the expression levels of ERS markers (GRP78, ATF4, p‐IRE1, and CHOP) were decreased by transfecting of NUP85‐siRNA, and the expression levels of these ERS markers were increased by transfecting of pcDNA3.1‐3‐NUP85 in TGF‐β1‐induced LX‐2 cells (Figure 4D–F; Figure S11B–G). Collectively, these results demonstrated that ERS in liver fibrosis was regulated by NUP85.

Next, the relationship between NUP85‐mediated liver fibrosis and ERS was explored. Control and NUP85 knockdown mice with CCl_4_‐induced liver fibrosis were treated with vehicle or tunicamycin (TM, a pharmacological activator of ERS). Western blotting results demonstrated that TM treatment significantly elevated the expression levels of ERS markers in liver tissues of mice, especially in NUP85 knockdown mice (Figure 4G; Figure S11H). Meanwhile, Western blotting and IHC results revealed TM administration induced marked upregulation of fibrosis markers in NUP85 knockdown mice (Figure 4H,P; Figure S11I,Q). Serological analysis results revealed TM administration exacerbated liver injury in NUP85 knockdown mice, as evidenced by elevated ALT, AST, and ALP expression levels (Figure 4I–K; Figure S11J–L). Additionally, H&E staining results confirmed TM‐treated hepatocyte necrosis was significantly augmented in NUP85 knockdown mice (Figure 4O; Figure S11P). Masson staining, Sirius Red staining, and hydroxyproline quantification results confirmed TM treatment increased collagen deposition in NUP85 knockdown mice (Figure 4L–O; Figure S11M–P). These findings demonstrated ERS may serve as a critical mediator of NUP85‐regulated liver fibrosis.

### 2.5 NUP85 Regulates ERS in HSCs by Modulating the ASK1‐MAPK Signaling Pathway

**FIGURE 5:**
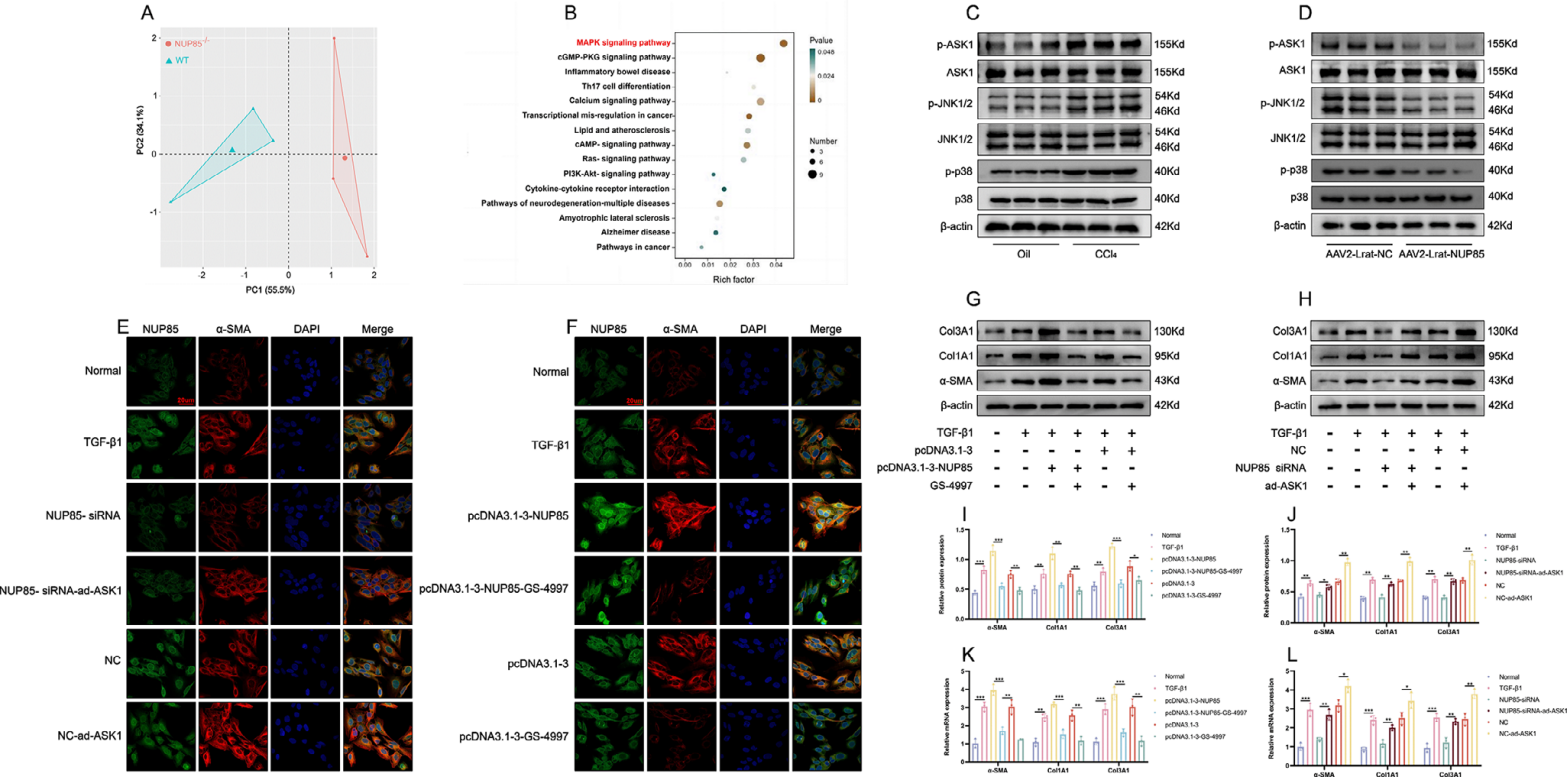
NUP85 knockdown mediates ERS through the ASK1 signaling cascade, thereby alleviating liver fibrosis. (A) Principal component analysis of NUP85 knockdown mice and blank control mice liver RNA‐seq results. (B) KEGG analyzes signaling pathways that undergo significant changes in biological processes. (C, D) Expression levels of ASK1, p‐ASK1, JNK1/2, p‐JNK1/2, p38, and p‐p38 in liver tissues of mice were detected by Western blotting. (E, F) Expression levels of NUP85 and α‐SMA in LX‐2 cells were detected by IF (scale bars, 20 µm). (G–L) Expression levels of α‐SMA, Col1A1, and Col3A1 in LX‐2 cells were detected by Western blotting and RT‐qPCR. All data are presented as the mean ± SD (n =3 independent experiments). Levels of statistical significance are indicated as ^*^ *p* < 0.05, ^**^ *p* < 0.01, ^***^ *p* < 0.001. One‐way ANOVA with Tukey test analysis and a two‐tailed Student *t* test were used for statistical analysis.

To systematically demonstrate the function role of NUP85 in liver fibrosis, RNA‐seq results were further analyzed. Cluster analysis result confirmed that the gene expression patterns were significantly different between control and NUP85 knockdown mice (Figure 5A). KEGG analysis result showed that the mitogen‐activated protein kinase (MAPK) signaling pathway was the most significantly altered signaling pathway when NUP85 was knockdown, suggested that NUP85 may regulate the progression of liver fibrosis through the MAPK signaling pathway (Figure 5B). Subsequent investigation focused on the regulatory role of NUP85 in the MAPK signaling pathway. The JNK/p38 signaling pathway was significantly activated, whereas knockdown of NUP85 significantly inhibited the activation of this signaling pathway in the liver tissues of CCl_4_‐induced mice (Figure 5C,D). Similarly, transfection of NUP85‐siRNA inhibited the JNK/p38 signaling pathway, whereas transfection of pcDNA3.1‐3‐NUP85 activated the JNK/p38 signaling pathway in TGF‐β1‐induced LX‐2 cells (Figure S12E–H). ASK1 is a member of the MAPK signaling pathway family that activates the downstream JNK/p38 signaling pathway, and previous studies have shown that ASK1 regulates ERS in the liver [16, 17]. Therefore, the effect of NUP85 on ASK1 was evaluated. The results of Western blotting revealed that knockdown of NUP85 inhibited ASK1 activation and overexpression of NUP85 promoted ASK1 activation (Figure 5C,D; Figure S12A–H). Taken together, these results suggested that NUP85 predominantly regulates the ASK1‐JNK/p38 signaling pathway in HSCs.

To determine the requirement of ASK1 activity in NUP85‐mediated regulation of HSCs activation, LX‐2 cells were treated with adenoviral‐mediated ASK1 overexpression (Ad‐ASK1) or the ATP‐competitive inhibitor GS‐4997. Western blotting results demonstrated that ASK1 activation was significantly enhanced by Ad‐ASK1 and inhibited by GS‐4997 in LX‐2 cells (Figure S12I–L). To mechanistically investigate the function role of ASK1‐dependent NUP85, Ad‐ASK1 was transfected, while knockdown of NUP85 and GS‐4997 was treated while overexpressing NUP85 in TGF‐β1‐induced LX‐2 cells. Western blotting, RT‐qPCR, and IF results revealed that ASK1 overexpression rescued NUP85 knockdown‐induced suppression of HSCs activation, conversely, ASK1 inhibition abrogated NUP85 overexpression‐driven pro‐activation effects (Figure 5E–L; Figure S13I–L). Furthermore, NUP85‐dependent regulation of ERS was demonstrated to operate through the ASK1‐JNK/p38 signaling pathway (Figure S113A–H). Thus, these data suggested that ASK1 is an indispensable target of NUP85 in the regulation of liver fibrosis.

### 2.6 NUP85 Binds to Deubiquitinating Enzyme USP47

**FIGURE 6:**
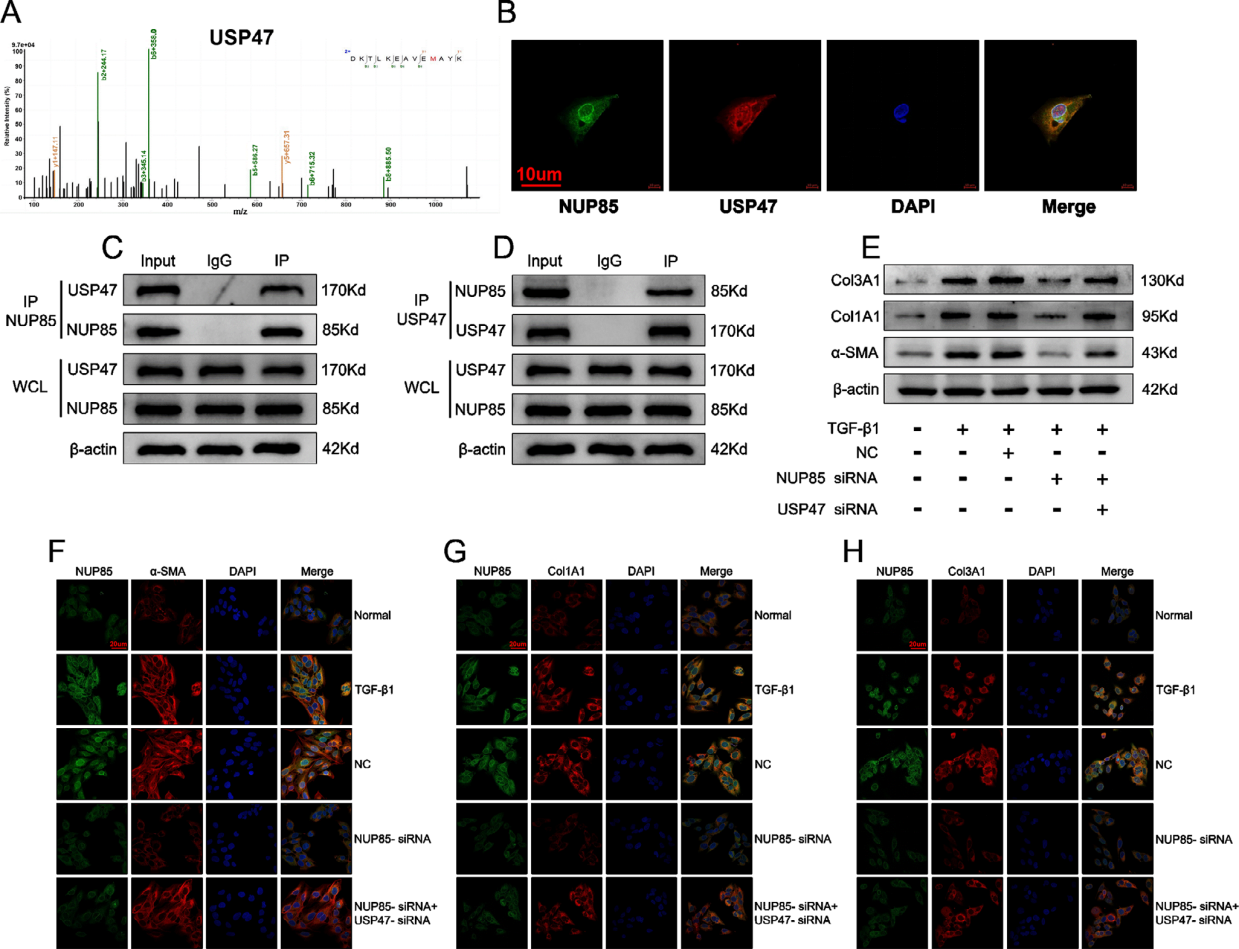
NUP85 regulates ERS and liver fibrosis through binding to USP47. (A) Mass spectrometry results revealed USP47 as a potential binding protein for NUP85. (B) IF result revealed localization of NUP85 and USP47 in LX‐2 cells. (C, D) CO‐IP results revealed the interaction of NUP85 with USP47 in LX‐2 cells. (E) Expression levels of α‐SMA, Col1A1, and Col3A1 in LX‐2 cells were detected by Western blotting. (F–H) Expression levels of NUP85, α‐SMA, Col1A1, and Col3A1 in LX‐2 cells were detected by IF (scale bars, 20 µm). All data are presented as the mean ± SD (n =3 independent experiments). One‐way ANOVA with Tukey test analysis and a two‐tailed Student t test were used for statistical analysis.

To elucidate the regulatory mechanism of NUP85 on ASK1 activity, the ubiquitination and phosphorylation levels of ASK1 were examined. Previous studies have demonstrated that ASK1 ubiquitination is closely associated with its activity. Consistent with prior reports, this study revealed increased ubiquitination and phosphorylation levels of ASK1 in liver fibrosis (Figure S14A). Furthermore, NUP85 was found to regulate ASK1 ubiquitination and phosphorylation levels (Figure S14B,C). Subsequently, TAK‐243 (an E1 enzyme activator inhibitor that suppresses de novo ubiquitination) was shown to reverse NUP85's effects on ASK1 ubiquitination and phosphorylation levels (Figure S14D). Notably, phosphorylation inhibitors (sorafenib and sunitinib, two broad‐spectrum protein kinase inhibitors) did not affect NUP85's regulation of ASK1 ubiquitination and phosphorylation levels (Figure S14E,F). These data indicated that NUP85 regulates ASK1 phosphorylation levels via ubiquitin modification, thereby affecting its activity. Subsequently, we screened ubiquitin‐associated enzymes from NUP85‐IP‐MS, NUP85‐RNA‐seq, and ASK1‐IP‐MS data (Figure S14G). USP47 was identified as a candidate protein (Figure 6A). IF results showed a considerable degree of co‐localization for NUP85 and USP47 in LX‐2 cells (Figure 6B). Coimmunoprecipitation (CO‐IP) results confirmed direct physical interaction between NUP85 and USP47 (Figure 6C,D). Subsequent IF results revealed that the expression level of USP47 was significantly reduced in TGF‐β1‐induced LX‐2 cells (Figure S15A). In addition, Western blotting, RT‐qPCR, and IHC results demonstrated that the expression level of USP47 was decreased in CCl_4_‐induced mice and increased in NUP85 knockdown mice (Figure S15B–L). The same phenomenon was observed in primary HSCs (Figure S15M–U). These results suggested that NUP85 interacts with the deubiquitinating enzyme USP47 and regulates the expression level of USP47.

To further verify whether USP47 mediates the role of NUP85 in liver fibrosis, co‐transfection of NUP85‐siRNA and USP47‐siRNA was conducted in TGF‐β1‐induced LX‐2 cells. Western blotting, RT‐qPCR, and IF results showed that the expression level of USP47 in the dual‐transfection group exhibited a marked reduction compared to the NUP85‐siRNA single‐transfection group (Figure S16A–C). Additionally, the expression levels of α‐SMA, Col1A1, and Col3A1 were higher in the co‐transfected group compared to the NUP85‐siRNA single‐transfection group (Figure 6E–H). Similarly, ERS markers (GRP78, ATF4, p‐IRE1, and CHOP) also showed higher expression levels in the co‐transfected group (Figure S16D–H). Thus, these results suggested that NUP85 regulates LX‐2 cells activation and ERS through binding to USP47.

### 2.7 NUP85 Regulates ASK1 through USP47‐Mediated Ubiquitination Modification

**FIGURE 7:**
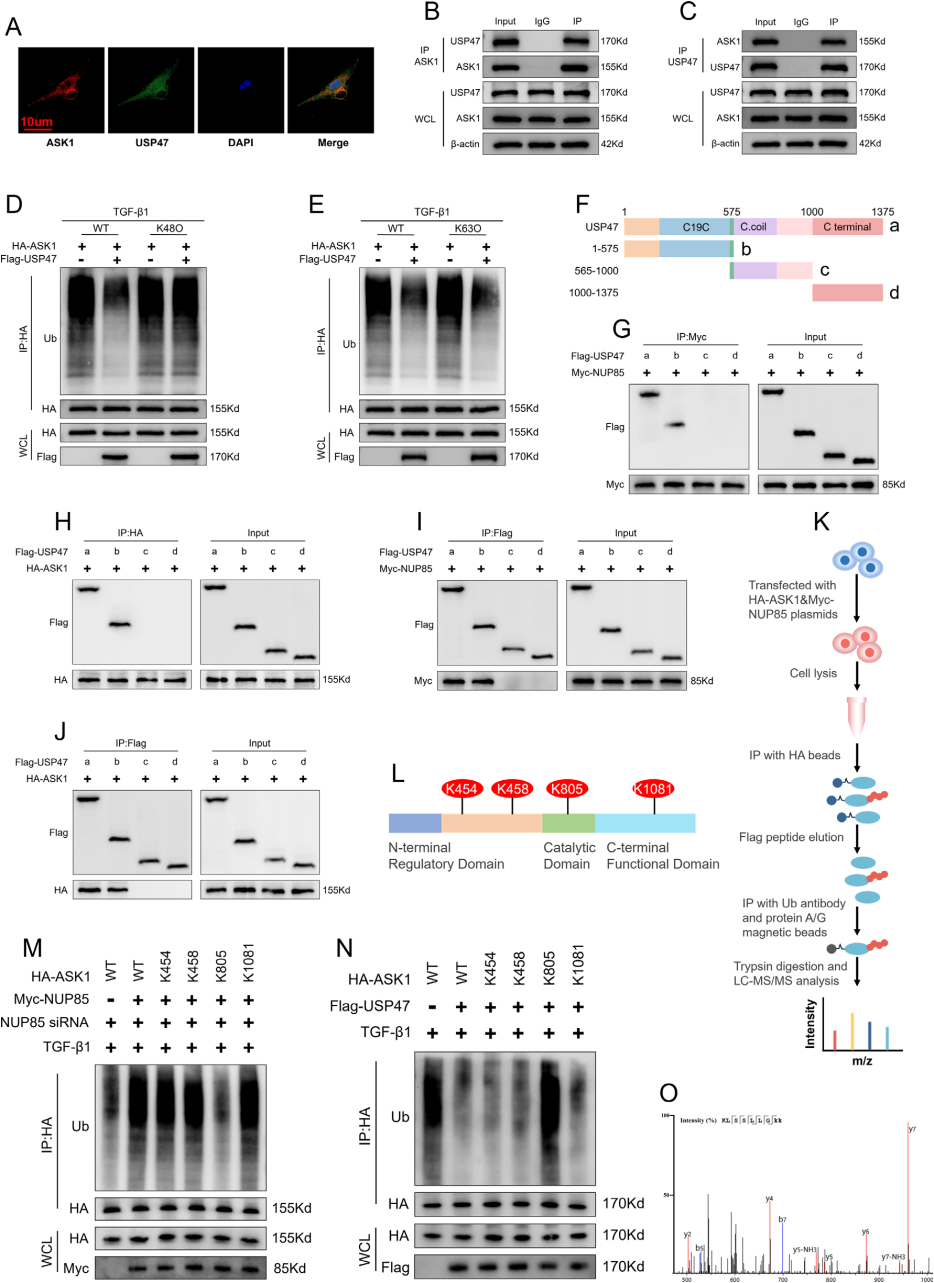
NUP85 regulates ubiquitination of ASK1 through binding to USP47. (A) IF result revealed localization of ASK1 and USP47 in LX‐2 cells. (B, C) CO‐IP results revealed the interaction of ASK1 with USP47 in LX‐2 cells. (D, E) Ubiquitination level of HA‐ASK1 was detected by Western blotting in LX‐2 cells. (F) Schematic representation of the structural domain of USP47. (G‐J) CO‐IP was used to detect the structural domains of NUP85 and ASK1 bound to USP47 in 293T cells. (K) Schematic of the workflow for identifying the ubiquitination sites of ASK1 by mass spectrometry analysis. (L) Four potential lysine ubiquitination sites of ASK1 were identified by mass spectrometry analysis. (M, N) Ubiquitination level of HA‐ASK1 was detected by Western blotting in LX‐2 cells. (O) Mass spectrometry identification of K805 ubiquitination of ASK1. All data are presented as the mean ± SD (n =3 independent experiments). One‐way ANOVA with Tukey test analysis and a two‐tailed Student t test were used for statistical analysis.

To determine whether USP47 functions as an E3 ligase mediating the ubiquitination modification of ASK1, the physical interactions between them were investigated. IF results showed a considerable degree of co‐localization for USP47 and ASK1 in LX‐2 cells (Figure 7A). Further CO‐IP results indicated a direct interaction between USP47 and ASK1 (Figure 7B,C). Furthermore, when co‐transfected with NUP85‐siRNA and USP47‐siRNA in TGF‐β1‐induced LX‐2 cells, the effect of NUP85 on ASK1 deubiquitination was inhibited (Figure S14H). These results confirmed that NUP85 mediates ubiquitination and phosphorylation activation of ASK1 through binding to USP47. Notably, USP47 overexpression did not alter K48‐linked ASK1 ubiquitination, consistent with published findings (Figure 7D). Subsequent analysis focused on K63‐linked ubiquitination modifications. Western blotting results revealed USP47 overexpression significantly suppressed K63‐linked ASK1 ubiquitination (Figure 7E). Notably, ASK1 exhibited dose‐dependent inhibition of NUP85‐USP47 binding, while reciprocal experiments demonstrated NUP85 similarly disrupted ASK1‐USP47 interaction in a dose‐dependent manner (Figure S14I,J). Thus, we speculated that NUP85 and ASK1 compete with each other for USP47 binding. To delve into the complex dynamics of protein‐protein interaction (PPI), truncated vectors targeting different structural domains of USP47 were designed (C19C, C.coil and C terminal) (Figure 7F), and CO‐IP result revealed that the C19C structural domain was the major linkage for competitive binding of NUP85 and ASK1 (Figure 7G–J).

Subsequently, we performed site‐specific ubiquitination mapping to identify ubiquitin conjugation sites on ASK1. Following immunoprecipitation of polyubiquitinated HA‐ASK1 complexes, LC‐MS/MS was utilized for precise site mapping (Figure 7K). Proteomic profiling identified four candidate ubiquitination sites at lysine residues K454, K458, K805, and K1081 (Figure 7L). To functionally characterize these sites, we constructed lysine‐to‐arginine substitution mutants (K454R, K458R, K805R, K1081R) using site‐directed mutagenesis. Systematic in vitro deubiquitination assays were subsequently conducted to determine which specific lysine residues mediate USP47‐dependent regulation of ASK1 ubiquitination. As shown in Figure 7M, recombinant expression of Myc‐NUP85 in NUP85 knockdown LX‐2 cells significantly increased the level of ubiquitination of wild‐type HA‐ASK1. Increased ubiquitination of ASK1 was also observed in the K454R, K458R, and K1081R mutants of HA‐ASK1, but not in the K805R mutant. Similarly, recombinant expression of Flag‐USP47 significantly reduced the ubiquitination level of wild‐type HA‐ASK1. Reduced ubiquitination of ASK1 was also observed in the K454R, K458R, and K1081R mutants of HA‐ASK1, but not in the K805R mutant (Figure 7N). Ubiquitination at K805 of ASK1 was independently validated by LC‐MS/MS, as shown in Figure 7O. Taken together, these data suggested that K805 was a major ubiquitination site of NUP85 mediated by USP47.

### 2.8 CREKA‐Lip/MV Alleviates Liver Fibrosis by Targeting NUP85 in Activated HSCs

To further investigate the therapeutic potential of NUP85 in liver fibrosis, a NUP85 inhibitor was obtained by virtual screening to demonstrate that inhibition of NUP85 could ameliorates liver fibrosis. Many plants have protective and therapeutic effects on liver fibrosis, and currently, nearly half of the drugs used in the treatment of liver fibrosis are natural product derivatives [18, 19]. Therefore, to identify novel NUP85 inhibitors, structure‐based virtual screening was performed using the Traditional Chinese Medicine Systems Pharmacology (TCMSP) Database. The results of the virtual screen suggested that MV may target and inhibit NUP85 (Figure S17A). Molecular docking simulations were performed to predict binding modes and thermodynamic parameters. Docking analysis result revealed stable occupation of the catalytic pocket in NUP85 by MV, with key hydrogen bonds formed between the pyranose hydroxyl groups of MV and the side chains of Glu67/Glu141 in NUP85 (Figure S17B). Additionally, CETSA was used to demonstrate that the ability of MV to target NUP85 (Figure S17C,D). To explore the pharmacological role of MV, flow cytometry and CCK8 were used to detect the effects of MV in LX‐2 cells. As shown in Figure S17E,F, 10∼80 uM of MV had no effect on the cellular activity of LX‐2 cells, which indicated that MV has good biosafety. Furthermore, MV reduced the expression levels of α‐SMA and Col1A1 in a dose‐dependent manner in TGF‐β1‐induced LX‐2 cells (Figure S17G). Thus, these results suggested that MV could target NUP85 and inhibit TGF‐β1‐induced LX‐2 cells activation.

To evaluate the therapeutic potential of MV against liver fibrosis, CCl_4_‐induced liver fibrosis mice were constructed. MV administration was initiated via oral gavage from week 5, with twice‐weekly dosing maintained for four weeks (Figure S18A). Serological analysis results demonstrated that the expression levels of ALT, AST, and ALP were reduced by MV treatment (Figure S18E–G). H&E staining result confirmed that CCl_4_‐induced liver parenchymal injury was attenuated by MV treatment (Figure S18B). Masson staining, Sirius red staining, and hydroxyproline quantification results indicated that CCl_4_‐induced collagen deposition was reduced by MV treatment (Figure S18B–D,H). Western blotting, RT‐qPCR, and IHC results revealed that downregulated expression levels of liver fibrosis markers (α‐SMA, Col1A1, and Col3A1) and NUP85 following MV treatment in CCl_4_‐induced mice (Figure S18B,I–K,N,O). Consistent with the mechanistic link between NUP85 and ERS in liver fibrosis, MV treatment significantly reduced the expression levels of ERS markers (GRP78, ATF4, p‐IRE1, and CHOP) and NUP85 in CCl_4_‐induced mice (Figure S18L,M,P,Q). Thus, these results suggested that MV could target NUP85 and ameliorate CCl_4_‐induced liver fibrosis.

**FIGURE 8:**
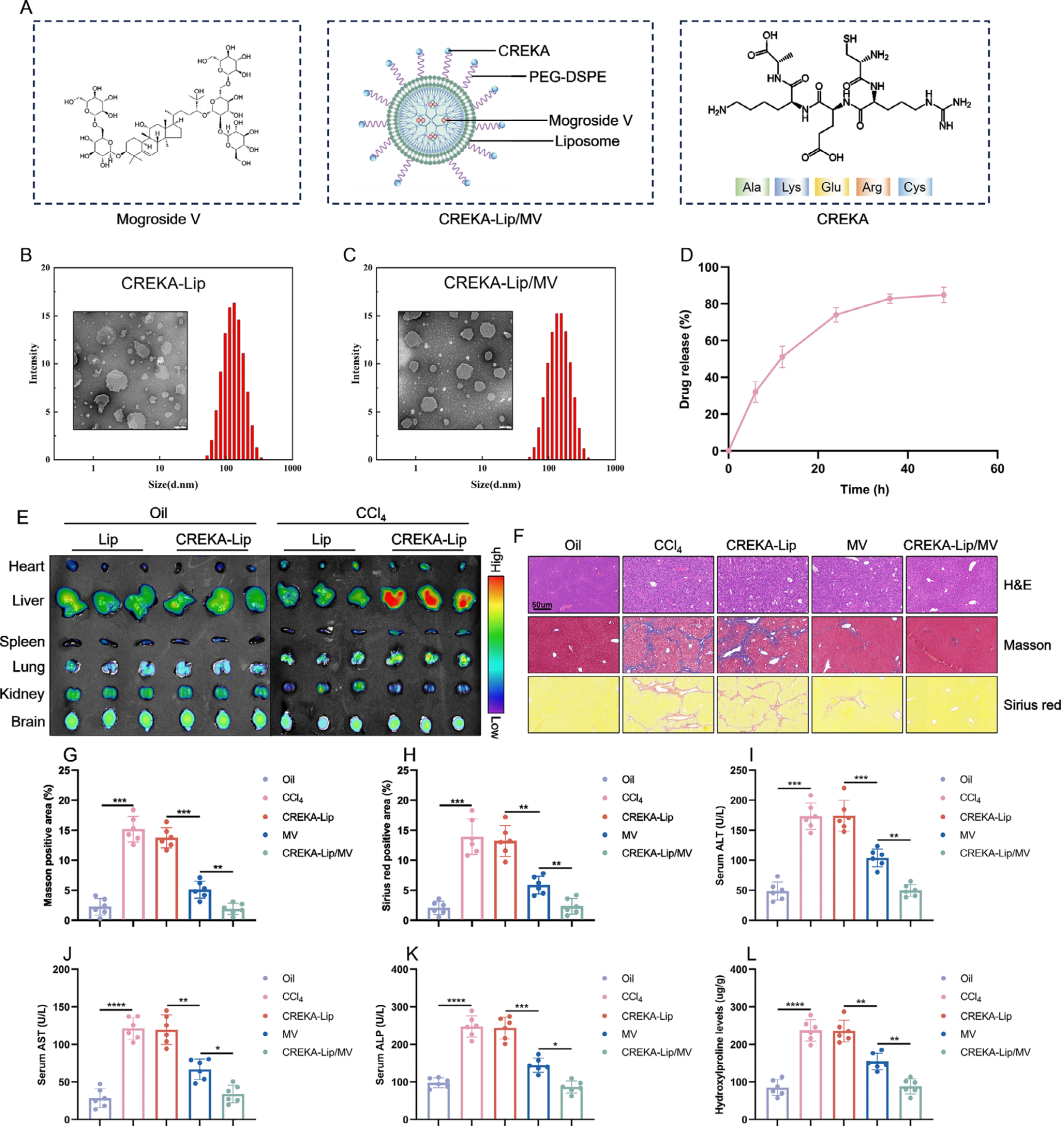
CREKA‐Lip/MV effectively attenuates CCl_4_‐induced liver fibrosis. (A) Schematic illustration of MV, CREKA, and CREKA‐Lip/MV. (B, C) Representative TEM images and dynamic light scattering data for CREKA‐Lip and CREKA‐Lip/MV. (D) In vitro release of sorafenib from CREKA‐Lip/MV in PBS medium containing 0.2% Tween‐80. (E) In vivo imaging of organs stripped from mice after treatment with DiD‐labeled liposomes (n=3). (F‐H) Representative H&E staining, Masson staining, and Sirius red staining images, Masson positive area (%), and Sirius red positive area (%) in liver tissues of mice (scale bars, 50 µm. n=6). (I‐K) Serum expression levels of ALT, AST, and ALP in mice (n=6). (L) Liver hydroxyproline content in mice (n=6). All data are presented as the mean ± SD (n =3 independent experiments). Levels of statistical significance are indicated as ^*^ *p* < 0.05, ^**^ *p* < 0.01; ^***^ *p* < 0.001, ^****^ *p* < 0.0001. One‐way ANOVA with Tukey test analysis and a two‐tailed Student *t* test were used for statistical analysis.

To improve therapeutic targeting of MV to liver fibrosis lesions, CREKA peptides bound to fibronectin were used to functionalize PEGylated liposomes for specific delivery of activated HSCs. The structural design of CREKA peptide‐conjugated liposomes encapsulating MV (CREKA‐Lip/MV) was presented in Figure 8A. Hydrodynamic analysis result showed similar particle sizes between CREKA‐Lip and CREKA‐Lip/MV, with average diameters of 110 nm and polydispersity indices below 0.2 (Figure 8B,C). TEM images confirmed spherical shapes for both formulations (Figure 8B,C). In vitro release studies demonstrated sustained MV release from CREKA‐Lip/MV, reaching over 80% cumulative release after 48 h (Figure 8D).

To investigate whether modification of CREKA could promote liposome‐targeted activation of HSCs, the fluorescent dye DiD was used to label Lip and CREKA‐Lip to monitor the uptake process in LX‐2 cells. IF results showed that the fluorescence signal were significantly increased treated with DiD‐labeled CREKA‐Lip compared to treated with DiD‐labeled Lip in LX‐2 cells (Figure S19D). In competition experiments, cellular uptake of CREKA‐Lip was markedly impaired in LX‐2 cells pretreated with excess free CREKA, further demonstrating the necessity of CREKA modification for targeting activated HSCs (Figure S19D). To investigate the properties of CREKA‐Lip for targeting activated HSCs in vivo, in vivo imaging was used to assess the biodistribution of DiD‐labeled Lip and CREKA‐Lip. In Oil‐treated mice, both Lip and CREKA‐Lip showed comparable fluorescence distribution across major organs. In CCl_4_‐induced mice, Lip maintained distribution patterns similar to Oil‐treated mice, while CREKA‐Lip exhibited significantly enhanced liver accumulation vs. Lip (Figure 8E). Following confirmation of activated HSCs targeting, the anti‐fibrosis effects of CREKA‐Lip/MV through inhibiting NUP85 were subsequently evaluated. First, the role of CREKA‐Lip/MV was investigated in CCl_4_‐induced mice. Serological analysis results showed that CREKA‐Lip/MV reduced the expression levels ALT, AST, and ALP better than free MV in CCl_4_‐induced mice (Figure 8F–H). H&E staining result showed that CREKA‐Lip/MV better alleviated liver injury compared to free MV (Figure 8J). The results of Masson staining, Sirius Red staining, and hydroxyproline quantification showed that CREKA‐Lip/MV better inhibited collagen deposition compared to free MV (Figure 8I,J). Similarly, IHC and Western blotting results showed that CREKA‐Lip/MV reduced the expression levels of NUP85, α‐SMA, Col1A1, and Col3A1 better compared to free MV (Figure S19A,B). Western blotting and IF results showed that CREKA‐Lip/MV reduced the expression levels of NUP85, α‐SMA, Col1A1, and Col3A1 better compared to free MV in TGF‐β1‐induced LX‐2 cells (Figure S19C,E–G). To assess the biosafety of CREKA‐Lip and CREKA‐Lip/MV, CCK‐8 was used to detect the cellular activity of LX‐2 cells. The result of CCK‐8 showed that no significant change in cellular activity was detected after treatment with CREKA‐Lip or CREKA‐Lip/MV compared to treatment with PBS in LX‐2 cells. (Figure S20A). In addition, the result of H&E staining showed that after treatment with CREKA‐Lip or CREKA‐Lip/MV, the major organs (heart, liver, spleen, lungs, and kidneys) of the mice retained their intact organ structures without pathological changes compared to treatment with PBS (Figure S20B) Thus, these data suggested that liver fibrosis could be alleviated by CREKA‐Lip/MV with a favorable biosafety profile by targeting NUP85 in HSCs.

## 3 Discussion

The regression of liver fibrosis is achievable following the elimination of underlying pathogenic factors [20, 21]. Despite substantial advances in deciphering the mechanisms governing the initiation and reversal of liver fibrosis in recent decades, critical aspects of its molecular underpinnings remain incompletely characterized [22, 23, 24]. Emerging evidence has elucidated the role of NUP85 in various liver pathologies. For instance, utilizing MCD diet‐induced mice and FFA‐exposed AML‐12 cells, demonstrated that NUP85 alleviates live lipid accumulation and inflammation in NAFLD by modulating the PI3K/AKT signaling pathway [11]. Separately, another research focusing on chaperone‐mediated autophagy (CMA) in liver macrophages revealed that CMA impairment‐induced NUP85 degradation exacerbates monocyte recruitment, thereby accelerating NAFLD progression [25, 26, 27]. Nevertheless, the specific functions and mechanistic basis of NUP85 in liver fibrosis remain poorly defined. This study investigates the role of NUP85 in liver fibrosis. It clarified that the progression of liver fibrosis is regulated by NUP85 in HSCs, rather than in hepatocytes or macrophages. Mechanistically, NUP85 binds to the deubiquitinating enzyme USP47. This interaction facilitates the ubiquitination of ASK1 at lysine residue 805 and enhances its subsequent phosphorylation‐dependent activation, thereby promoting ERS and ultimately driving the progression of liver fibrosis. Furthermore, Mogroside V (MV) was identified as a pharmacological inhibitor of NUP85 and engineered CREKA peptide‐conjugated liposomes encapsulating MV (CREKA‐Lip/MV) to target activated HSCs, thereby inhibiting the progression of liver fibrosis.

ERS as a key factor regulating the progression of liver fibrosis, it is a pathological process of endoplasmic reticulum physiological function disorders caused by the disruption of endoplasmic reticulum Ca^2+^ homeostasis or protein processing and transportation disorders [28, 29]. ERS promotes the development of liver fibrosis by inducing apoptosis in hepatocytes, and it induces the activation of the apoptotic pathway through the regulation of myoendoplasmic reticulum Ca^2+^ ATPase and Ca^2+^ homeostasis [30, 31]. Additionally, since HSCs are rich in endoplasmic reticulum and secreted proteins, they are more sensitive to alterations in endoplasmic reticulum homeostasis, and it has been reported that ERS can induce HSCs activation through the IRE1‐Xbp1 signaling pathway [32, 33]. Inhibition of ERS is essential for hepatocyte survival and HSCs inactivation in liver fibrosis. NUP85 is associated with the regulation of ERS. Our results demonstrated that knockdown of NUP85 suppressed ERS and thus HSCs activation, leading to amelioration of liver fibrosis. However, it has also been shown that ERS induces apoptosis in HSCs, thereby favoring the attenuation of liver fibrosis. This is inconsistent with our findings and those of other studies and may be due to the fact that ERS regulates the function of HSCs through different mechanisms in different environments or diseases.

The MAPK signaling pathway is mainly composed of extracellular signal‐regulating enzymes, protein regulatory kinase p38, and c‐Jun end‐regulated kinase [34, 35]. Existing research indicates that the MAPK signaling pathway contributes to liver fibrosis by activating HSCs [36, 37]. Our analysis pinpointed this signaling pathway as the principal mediator of NUP85‐regulated ERS in fibrotic progression. Aberrant JNK/p38 activation mediated through upstream ASK1 signaling has been shown to trigger both ERS and HSC activation during fibrotic pathogenesis [38]. However, the cellular consequences of JNK/p38 stimulation exhibit temporal and intensity‐dependent variations. This mechanistic understanding has shifted therapeutic focus toward ASK1 modulation in clinical interventions. Notably, there are studies suggesting that ASK1 may modulate endoplasmic reticulum stress [39, 40]. Here, we elucidated that NUP85 regulates endoplasmic reticulum stress in liver fibrosis through ASK1 and established a link between NUP85 function and MAPK signaling pathway activation. Importantly, the molecular targeting of ASK1 has been validated as a therapeutically promising strategy in hepatopathy management. In physiological homeostasis, ASK1 maintains an autoinhibited conformation through intramolecular interactions, preserving its structural quiescence. However, upon exposure to pathogenic stimuli, this stress‐activated kinase undergoes rapid post‐translational modification through phosphorylation events, transforming into a pivotal signaling node that orchestrates cellular stress‐response cascades.

The post‐translational modification mediated by ubiquitin conjugation exerts precise regulatory control over the functional status of ASK1 [41, 42]. E3 ubiquitin‐protein ligases, through their catalytic specificity, govern the topological configuration of polyubiquitin chains attached to ASK1 substrates [43]. This enzymatic discrimination mechanism ultimately dictates the molecular fate of ASK1, steering its activation states vs. proteasomal degradation pathways. Here, we used an unbiased approach to identify USP47 as a key factor in the regulation of ASK1 activity by NUP85 in liver fibrosis, and we found that USP47 inactivates the hyperactivated form of ASK1 by selectively removing polyubiquitination from this kinase. USP47 contains an N‐terminal C19 peptidase domain, a middle coil‐coiled domain, and a C‐terminus. N‐terminal C19 peptidase domain with a catalytic triad consisting of cysteine, histidine, and aspartic acid/asparagine is responsible for hydrolyzing the isopeptide bond between ubiquitin and the target protein. For example, USP47 interacts with SATB1 through the N‐terminal C19 peptidase domain and mediates its Lys48 ubiquitination. We found that in liver fibrosis, NUP85 mediates K63 polyubiquitination of ASK1 by competitively binding the N‐terminal C19 peptidase domain of UAP47 with ASK1. Notably, this deubiquitination simultaneously inhibited overactive ASK1 and its downstream signaling. Additionally, LC‐MS/MS analysis revealed that the ubiquitination levels of four lysine residues (K454, K458, K805, and K1081) in ASK1 were significantly altered by NUP85 knockdown. By constructing K454, K458, K805, and K1081 mutant plasmids, we found that the ubiquitination level of the K805 site affected ASK1 activation. Subsequent cellular experiments further verified that NUP85 regulates ASK1 activation by affecting the deubiquitination of K805 residues of ASK1 mainly through USP47. Future studies will focus on elucidating the specific molecular mechanisms by which NUP85 regulates ASK1 phosphorylation activation.

NUP85 overexpression exacerbates liver fibrosis and is accompanied by significant activation of ASK1 and ERS. In contrast, knockdown of NUP85 attenuated liver fibrosis and reduced activation of ASK1 and ERS. Therefore, inhibiting NUP85 and thereby reducing the activation of ASK1 and ERS may be a promising strategy to ameliorate liver fibrosis. We screened natural products that bind to NUP85 by molecular docking‐based virtual screening. We found that MV significantly inhibited the expression level of NUP85 and alleviated liver fibrosis, suggesting that pharmacological inhibition of NUP85 expression level is a promising strategy for clinical treatment of liver fibrosis. Notably, to improve the disadvantages of low bioavailability, poor stability, toxicity, and side effects of natural compounds, we modified PEGylated liposomes with CREKA to target activated HSCs in fibrosis liver. Notably, CREKA‐Lip/MV exhibits targeted effects on HSCs. HSCs not only regulate the progression of liver fibrosis but also play a crucial role in a range of liver diseases, including MASH, alcoholic liver disease (ALD), and HCC [44, 45, 46]. Therefore, CREKA‐Lip/MV not only alleviates the progression of liver fibrosis but also represents a significant therapeutic strategy for diseases characterized by HSCs activation as a core pathological process.

In summary, the present study confirms that NUP85 acts as a key mediator in the progression of liver fibrosis by regulating ASK1‐mediated ERS. Mechanistically, NUP85 facilitates the ubiquitylation of lysine residue 805 in ASK1 by binding to the deubiquitylating enzyme USP47 and increases its phosphorylation activation, resulting in the promotion of ERS. Additionally, we discovered NUP85 small molecule inhibitor‐MV and constructed CREKA‐Lip/MV to target activated HSCs resulting in inhibition of liver fibrosis progression. Therefore, targeting the NUP85‐USP47‐ASK1 signaling pathway may be a promising approach for the treatment of liver fibrosis.

## 4 Experimental Section

### 4.1 Materials

Male C57BL/6J mice were acquired from Cavins Laboratory Animal Co., Ltd. (Changzhou, China). Adeno‐associated virus serotype 8 (AAV8) and AAV2 vector systems were sourced from General Bio Co., Ltd. (Anhui, China). Cell culture reagents, including DMEM medium supplemented with 10% fetal bovine serum and 10 U/ml penicillin‐streptomycin, were procured from Gibco (Carlsbad, CA, USA). Collagenase type IV and streptavidin E were supplied by Macklin (Shanghai, China). RIPA lysis buffer and DAPI staining solution were provided by Beyotime (Shanghai, China). TRIzol reagent and SYBR Green were obtained from Cwbio (Suzhou, China). PBS‐T washing buffer was acquired from Thermo Fisher (Shanghai, China). Commercial assay kits for determining ALT, AST, ALP, and hydroxyproline concentrations were purchased from Nanjing Jiancheng Bioengineering Institute (Nanjing, China). All remaining analytical‐grade chemicals were sourced from Solarbio (Beijing, China).

### 4.2 Animal Models

Mice were housed under specific pathogen‐free (SPF) conditions, maintained at 22°C–24°C with 55%–60% relative humidity and subjected to a 12‐h light/dark cycle. Food and water were provided ad libitum. For the hepatic fibrosis model establishment, intraperitoneal injections of CCl_4_ were administered twice weekly over 8 weeks. The CCl_4_ solution was prepared at 20% concentration in olive oil and delivered at a dosage of 5 mL/kg body weight. Alternatively, mice received BDL surgery and were euthanized 2 weeks later. Blood and liver tissue were collected. The complete experimental protocol was performed at Anhui Medical University in compliance with animal welfare guidelines, following review and approval by the Institutional Animal Care and Use Committee of Anhui Medical University (Approval No. 2023‐N(A)‐99).

### 4.3 Human Samples

The clinical specimens collected in the study were obtained from wax blocks of cirrhotic and normal liver tissues from the Department of Pathology of the first hospital affiliated to the University of Science and Technology of China. Among them, 6 cirrhotic specimens were obtained from patients who underwent liver transplantation in the Department of Liver Transplantation; 6 normal liver tissues were obtained from patients who underwent hepatic hemangioma surgery in the Department of General Surgery. These tissues specimens were paraffin‐embedded and sectioned by the Department of Pathology and pathologically diagnosed. The pathological changes in all 12 specimens were in accordance with the clinical diagnosis, while the tissues 2 cm away from the edge of the hepatic hemangioma was diagnosed to be normal liver tissues without other pathological changes. This experiment was approved by the Ethics Committee of the first hospital affiliated to the University of Science and Technology of China (No. 2023KY413).

### 4.4 AAV8 Mediated Liver‐Specific NUP85 Knockdown and Overexpression

The hepatotropic AAV8 vector system for liver‐specific NUP85 gene silencing and overexpressing was custom‐developed, with AAV8‐NC serving as the scramble sequence control. Experimental cohorts received a single intravenous bolus (100 µL) of viral suspension through tail vein delivery, containing a standardized viral load quantified at 2 × 10^11^ genome copies per dose.

### 4.5 AAV2 Mediated HSCs‐Specific NUP85 Knockdown and Overexpression

The hepatotropic AAV2‐Lrat vector system for HSCs‐specific NUP85 gene silencing and overexpressing was custom‐developed, with AAV2‐NC serving as the scramble sequence control. Experimental cohorts received a single intravenous bolus (100 µL) of viral suspension through tail vein delivery, containing a standardized viral load quantified at 2 × 10^11^ genome copies per dose.

### 4.6 Cell Culture

The human hepatic stellate cell line LX‐2 was maintained in the School of Pharmaceutical Sciences at Anhui Medical University. Cells were cultured in Dulbecco's Modified Eagle Medium (DMEM), supplemented with 10% (v/v) fetal bovine serum and a combination of penicillin‐streptomycin antibiotics at a concentration of 10 U/ml. Six‐well culture plates received cellular suspensions adjusted to 1 × 10^6^ cells per well. Cellular activation involved 24 h exposure to transforming TGF‐β1. Rodent HSCs isolation implemented collagenase type IV and streptavidin E co‐perfusion methodology. Liver tissues digestion combined with enzymatic treatment with mechanical dissociation to yield individual cell suspensions. Density‐based fractionation utilizing Nycodenz gradient media was conducted according to established commercial protocols. Isolated HSCs were propagated in serum‐supplemented DMEM containing 1% penicillin‐streptomycin mixture under controlled incubation parameters (37°C, 5% carbon dioxide, 95% relative humidity).

### 4.7 H&E Staining, Masson Staining, and Sirius Red Staining

Liver tissue samples underwent immersion fixation in 10% neutral buffered formalin for 12–16 h at 4°C. Tissue processing involved sequential solvent exchange commencing with ethanol gradients and concluding with xylene clearing. Paraffin embedding was subsequently performed, followed by microtome sectioning at 4 µm thickness. After dewaxing and rehydration through descending alcohol series, histological staining was conducted using H&E staining, Masson staining, and Sirius Red staining according to standardized protocols. Microscopic evaluation was carried out with a digital imaging‐equipped light microscope for histopathological analysis.

### 4.8 Western Blotting

Cellular and liver specimens underwent mechanical disruption in RIPA lysis buffer supplemented with multi‐proteolytic enzyme blockers. Post‐lysis protein separation was achieved through discontinuous SDS‐PAGE electrophoresis, followed by membrane transfer for immunodetection using specified primary antibodies paired with horseradish peroxidase‐linked species‐matched secondary reagents. Antigen‐antibody complexes were visualized through enhanced chemiluminescence detection followed by digital image acquisition. The antibodies utilized for Western blot analysis are provided in Table S1.

### 4.9 Quantitative Real‐Time PCR

Liver RNA extraction from mice specimens were conducted with TRIzol reagent following standard protocols. Subsequently, 2 µg of purified RNA served as template for cDNA synthesis employing a commercial reverse transcription system. Quantitative real‐time PCR was performed using SYBR Green fluorescence detection, with GAPDH mRNA expression levels providing endogenous normalization. Transcript quantification was determined through comparative threshold cycle (2^−ΔΔCt^) calculation. The primer sequences used in this study are provided in Table S2.

### 4.10 Immunofluorescent (IF) Staining

IF analysis was conducted on mice liver tissue sections following established protocols. Liver specimens underwent paraffin embedding prior to microtome sectioning. Tissue sections underwent dewaxing via xylene treatment and gradual ethanol rehydration, followed by antigenic epitope recovery through sodium citrate‐mediated heat treatment (pH 6.0). Sections were immersed in PBS‐T buffer followed by 2 h serum blockade at ambient temperature. Primary antisera at predetermined titers were applied for 12 h incubation at 4°C, subsequently probed with species‐specific fluorochrome‐conjugated secondary reagents (60‐min incubation under light‐protected conditions). Nuclear counterstaining was achieved with DAPI (0.5 µg/mL) during mounting procedures for fluorescence microscopy observation. A complete list of all antibodies employed in this study, along with their corresponding dilution ratios, is detailed in Table S3.

### 4.11 Measurement of Serum Biochemical Indicators and Hepatic Hydroxyproline

Serum expression levels of ALT, AST, and ALP were quantified with standardized commercial detection systems following the protocols provided by the manufacturer. Liver tissues expression level of hydroxyproline was assessed through colorimetric analysis employing a specialized biochemical detection system as per standard operational guidelines.

### 4.12 Immunoprecipitation/Mass Spectrometry

Protein‐protein interaction (PPI) studies were conducted following established co‐immunoprecipitation protocols. LX‐2 cells received dual plasmid transfection over 24 h prior to harvesting. Mechanical cell disruption via sonication in NP40‐containing buffer facilitated lysate preparation for affinity purification. Antigen‐antibody complexes were immobilized overnight at 4°C using epitope‐specific magnetic beads targeting HA and Flag tags. Subsequent processing involved rigorous buffer washing cycles followed by protein denaturation at 95°C for 10 min in SDS‐containing solution. Immunodetection was achieved through sequential incubation with species‐specific immunoglobulin pairs. Proteomic characterization protocols included competitive displacement of HA‐NUP85 complexes using cognate peptide ligands, subsequent TCA‐mediated protein aggregation, and trypsin‐mediated proteolysis. Post‐digestion peptides underwent C18‐based hydrophobicity purification before chromatographic separation coupled with tandem mass spectrometry (LTQ platform). Spectral data interpretation employed SEQUEST software with UniProt‐curated reference sequences (https://www.uniprot.org/) for interaction partner validation.

### 4.13 Immunohistochemistry (IHC)

Liver specimens harvested from mice model underwent immediate immersion in neutral buffered formalin for 24 h fixation. Following standard paraffin‐embedding protocols, 5 µm histological sections were prepared for microscopic evaluation. Morphological characteristics were documented using bright‐field microscopy imaging systems. A comprehensive list of all antibodies utilized in this study, along with their respective dilution ratios, is documented in Table S4.

### 4.14 Cellular Thermal Shift Assay (CETSA)

Cellular populations were segregated into experimental cohorts: the treatment group receiving 80 µm MV stimulation for 24 h and the control group maintaining basal conditions. Following intervention, cell pellets were reconstituted in phosphate‐buffered saline (PBS) to achieve a density of 1 × 10^9^ cells/mL. The homogeneous suspension was aliquoted into six equivalent fractions for thermal stability profiling through incubation at graded temperatures (37°C–65°C, 10 min/condition). Post‐thermal treatment samples underwent lysis via high‐speed centrifugation (12 000 ×g, 30 min). The protein expression level of NUP85 was assessed by Western blotting.

### 4.15 Preparation and Characterization of Liposomes

DSPE‐PEG2000‐CREKA nanoconjugates were synthesized via thiol‐maleimide click chemistry, establishing covalent conjugation between the maleimide functionality of PEGylated phospholipids and sulfhydryl groups on CREKA peptides. This conjugation reaction was conducted in dimethyl sulfoxide solvent system with optimized stoichiometry (maleimide‐PEG2000‐DSPE: peptide = 10:8 molar ratio), maintained under light‐restricted conditions with continuous agitation at ambient temperature for 24 h. Purified conjugates were obtained through dialysis (MWCO 3.5 kDa) against deionized water, followed by lyophilization for cryopreservation at −20°C. Liposomal nanocarriers were fabricated via the thin‐film dispersion technique. For CREKA‐functionalized formulations (CREKA‐Lip), precisely weighed lipid components (S100 lipoid: cholesterol: DSPE‐PEG2000‐CREKA = 56:34:10 molar ratio) were co‐dissolved in chloroform/ethanol mixed solvent (2:1 v/v ratio). Rotary evaporation at 37°C under reduced pressure generated uniform lipid films, which underwent hydration with 5% glucose solution (1 mL) under controlled vortex flow. The resultant multilamellar vesicles were converted into monocellular structures through probe sonication (250 W output, 5 s on/off cycles, 2 min total duration). Control PEGylated liposomes (Lip) substituted the targeting ligand with methoxy‐PEG2000‐DSPE. For diagnostic/therapeutic cargo loading, DiD fluorescent probe or MV therapeutic agents were incorporated into the organic phase during film formation, maintaining precise mass ratios (MV: phospholipid = 1:16.56). Unincorporated payloads were eliminated via size‐exclusion chromatography using Sephadex G‐75 medium. Nanovesicle characterization included hydrodynamic diameter, surface charge, and size distribution (PDI) measurements by dynamic light scattering, complemented by transmission electron microscopy for morphological verification.

### 4.16 Statistical Analysis

All statistical analyses were performed using GraphPad Prism9.0. Data are presented as mean ± SD. The n value indicates the number of independent biological replicates or animals and is clearly labeled in each figure legend. For quantification, immunoblot signals were normalized to the corresponding loading controls, and fluorescence/intensity‐based assays were analyzed using identical acquisition settings and expressed as raw values or foldchange vs. the indicated control. No data transformation was applied, and no outliers were removed unless prespecified exclusion criteria were met or a technical failure occurred. Two‐group comparisons were performed using an unpaired two‐tailed Student's t‐test; comparisons among ≥3 groups used one‐way ANOVA with Tukey's post hoc test; and experiments with two independent variables used two‐way ANOVA with Sidak's multiple comparisons test. *P* < 0.05 was considered statistically significant.

## Author Contributions

D.S.Y., H.R.Y., L.X.P., and C.T. performed methodology and writing – original draft. D.S.Y., F.C.Z., S.L.H., X.R.L., and M.C. performed data curation and methodology. C.Q. performed the investigation. J.W. performed the investigation. Z.L.C. performed the methodology and writing – original draft. T.X. performed the investigation and writing – review & editing. All of the authors contributed to the final version of the manuscript.

## Conflicts of Interest

The authors declare no conflicts of interest.

## Supporting information




**Supporting File**: advs75024‐sup‐0001‐SuppMat.docx.

## Data Availability

The data that support the findings of this study are available from the corresponding author upon reasonable request.
