# Supplementary material for: NUP85 Mediates Endoplasmic Reticulum Stress through the USP47/ASK1 Signaling Pathway to Regulate the Progression of Liver Fibrosis
Source: Adv Sci (Weinh). 2026 Mar 28;13(33):e19972. doi: 10.1002/advs.202519972 (PMC13271639; doi:10.1002/advs.202519972)
Supplement: Supplementary file 1 — Supporting File: advs75024‐sup‐0001‐SuppMat.docx. [file ADVS-13-e19972-s001.docx]

Supporting Information

**NUP85 Mediates Endoplasmic Reticulum Stress through the USP47/ASK1 Signaling Pathway to Regulate the Progression of Liver Fibrosis**

*Dashuai Yang^1,†^, Haoran Yang^3,†^, Linxin Pan^1,4,†^, Chang Tian^1,†^, Fucheng Zuo^1^, Shilei Huang^1^, Xianrui Li^1^, Ming Chen^1^, Cheng Qian^5,*^, Jie Wang^1,2,*^, Zhaolin Chen^6,7,*^, Tao Xu**^1,2,*^*

**^1^**Inflammation and Immune Mediated Diseases Laboratory of Anhui Province, School of Pharmacy, Anhui Medical University, Hefei, 230032, China

**^2^**Department of Emergency Surgery, The Second Affiliated Hospital of Anhui Medical University, Hefei, 230032, China

**^3^**Anhui Institute of Medicine, Hefei, 230601, China

**^4^**School of Life Science, Anhui Medical University, Hefei, 230032, China

**^5^**Center for Scientific Research, Auhui Medical University, Hefei, 230032, China

**^6^**Department of Pharmacy, The First Affiliated Hospital of USTC, Division of Life Sciences and Medicine, University of Science and Technology of China, Hefei, Anhui, 230001, China

**^7^**Anhui Provincial Key Laboratory of Precision Pharmaceutical Preparations and Clinical Pharmacy, Hefei, Anhui, 230001, China

^⁎^Correspondence authors.

E-mail addresses: xutao@ahmu.edu.cn (T. Xu), czl0808@ustc.edu.cn (Z. Chen), wangjienar@ahmu.edu.cn (J. Wang), 2015510027@ahmu.edu.cn (C. Qian).

^†^These authors contributed equally to this work.

**
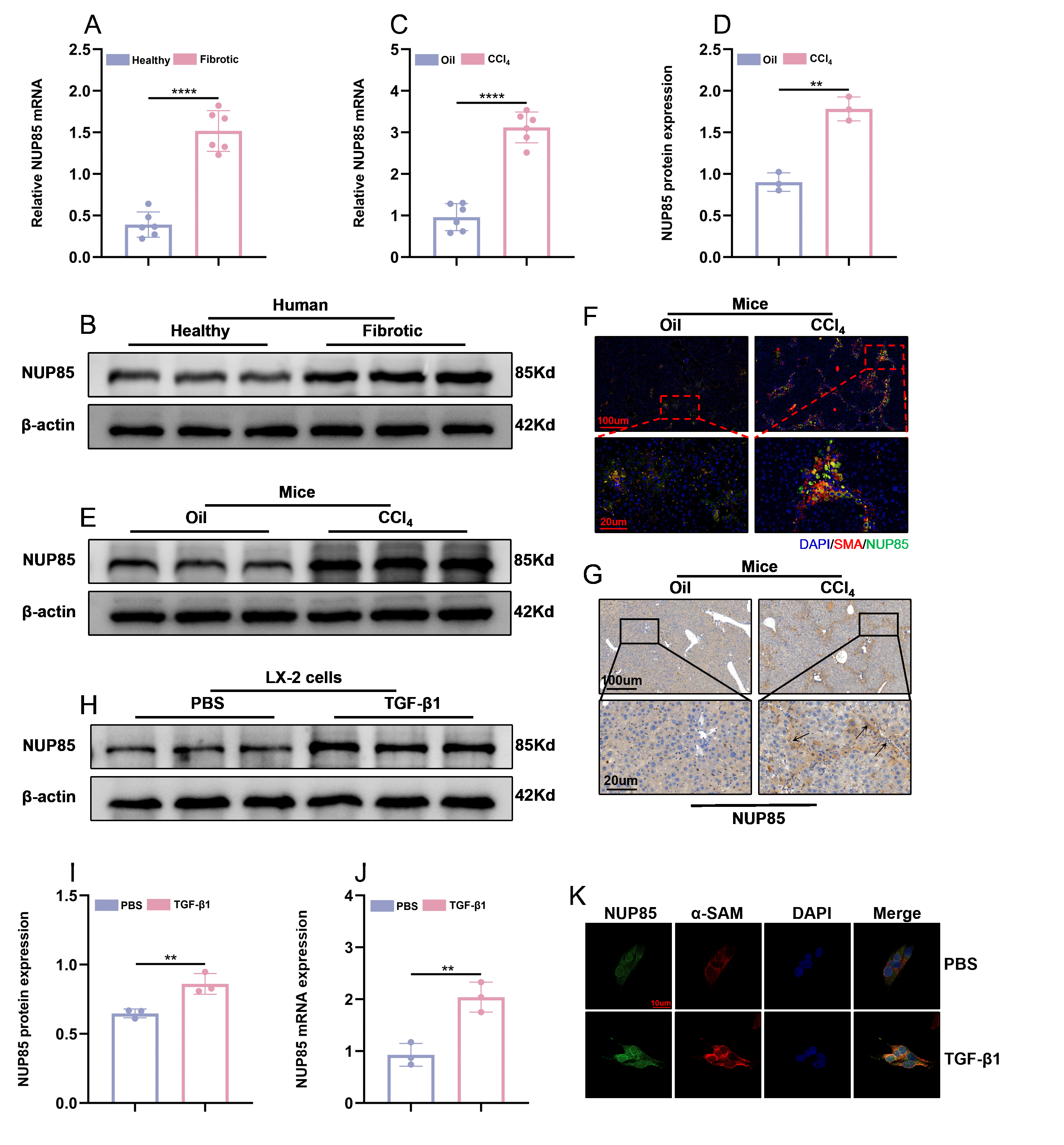
**

**Figure S1.** NUP85 expression level was elevated in liver fibrosis. (A, B) Expression level of NUP85 in liver tissues of human was detected by RT-Qpcr and Western blotting. (C) Expression level of NUP85 in liver tissues of mice was detected by RT-qPCR. (D, E) Expression level of NUP85 in liver tissues of mice was detected by Western blotting and bar plot. (F) Expression level of NUP85 in liver tissues of mice was detected IF (scale bars, 100µm, 20µm). (G) Expression level of NUP85 in liver tissues of mice was detected IHC (scale bars, 100µm, 20µm). (H, I) Expression level of NUP85 in LX-2 cells was detected by Western blotting and bar plot. (J, K) Expression level of NUP85 in LX-2 cells was detected by RT-qPCR and IF (scale bars, 10µm). All data are presented as the mean ± SD (n =3 independent experiments). Levels of statistical significance are indicated as ***P* < 0.01, *****P* < 0.0001. One‐way ANOVA with Tukey test analysis and a two‐tailed Student t test were used for statistical analysis.


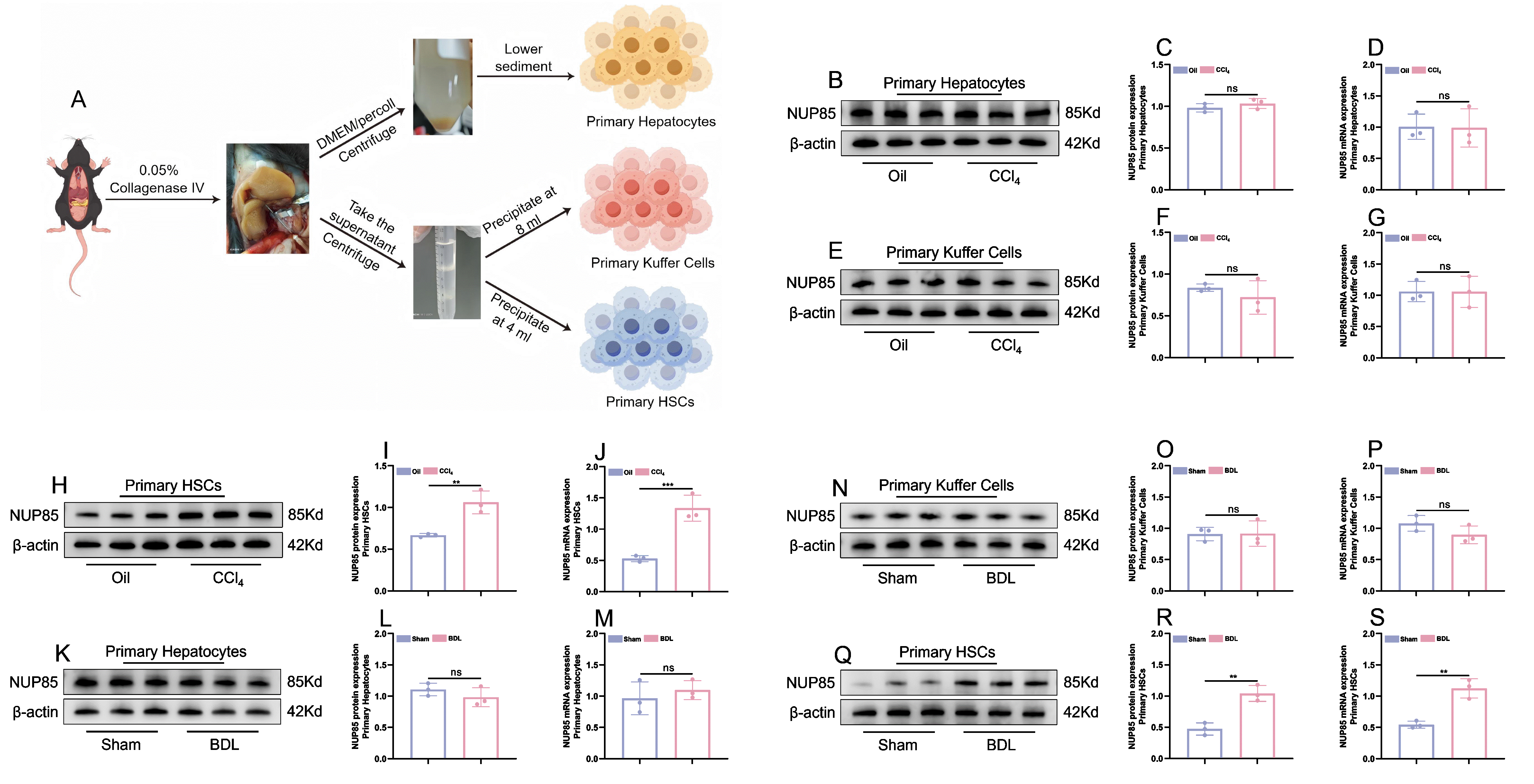


**Figure S2.** The expression level of NUP85 was detected in primary hepatocytes, primary Kuffer cells, and primary HSCs. (A) Schematic illustrates the process of isolating primary hepatocytes, primary Kuffer cells, and primary HSCs in mice. (B-J) The expression level of NUP85 was detected by Western blotting and RT-qPCR in primary hepatocytes, primary Kuffer cells and primary HSCs induced by CCl_4_. (K-S) The expression level of NUP85 was detected by Western blotting and RT-qPCR in primary hepatocytes, primary Kuffer cells and primary HSCs induced by BDL. All data are presented as the mean ± SD (n =3 independent experiments). Levels of statistical significance are indicated as **P < 0.01, ***P < 0.001, “ns” indicates no significance. One‐way ANOVA with Tukey test analysis and a two‐tailed Student t test were used for statistical analysis.

**
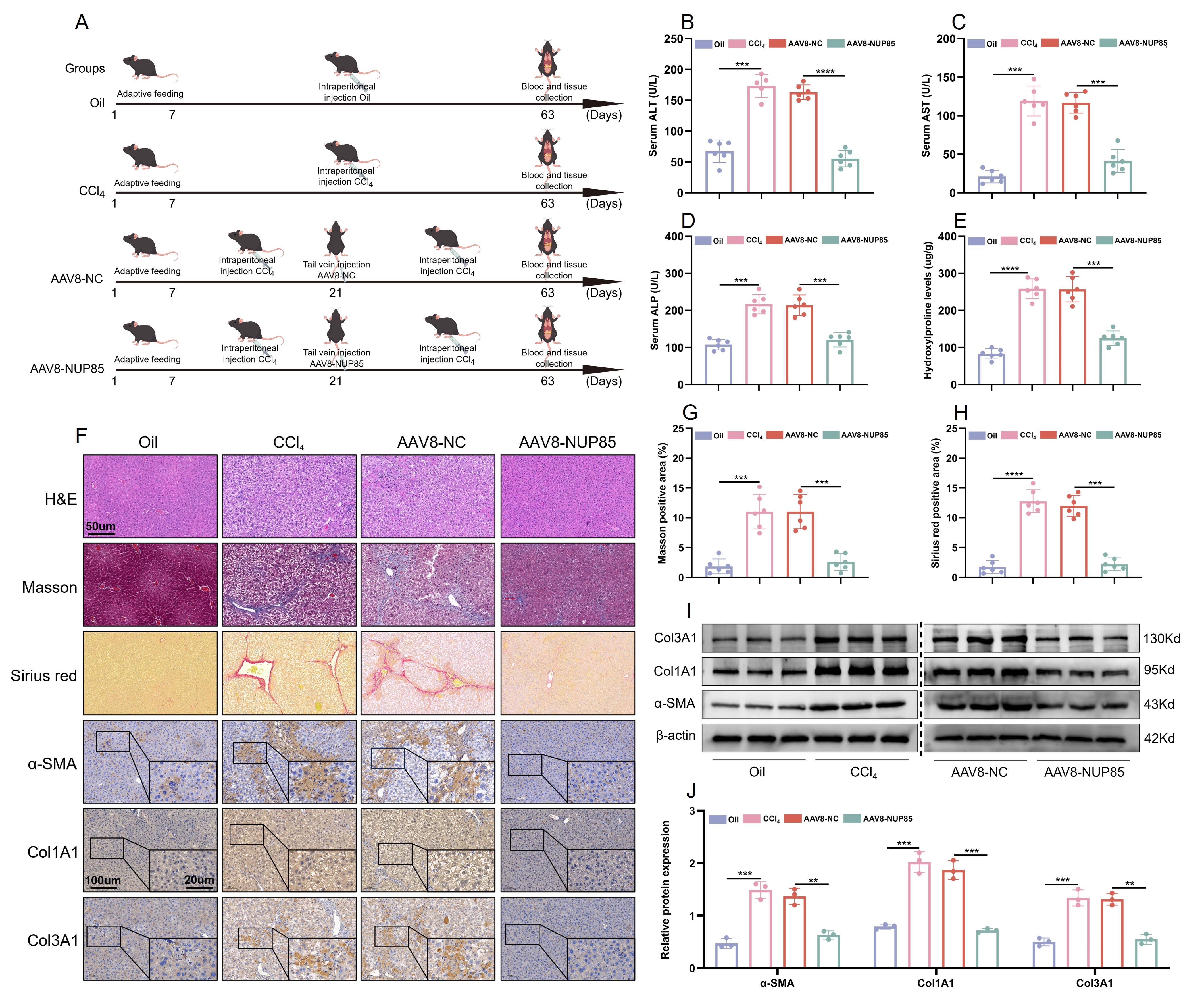
**

**Figure S3.** Liver-specific NUP85 knockdown alleviates CCl_4_-induced liver fibrosis. (A) Schematic illustrating of the experimental design of AAV8-NUP85 for the treatment of CCl_4_-induced liver fibrosis in mice (n=6). (B-D) Serum expression levels of ALT, AST and ALP in mice (n=6). (E) Liver hydroxyproline content in mice (n=6). (F-H) Representative H&E staining, Masson staining, Sirius red staining, and IHC images, Masson positive area (%) and Sirius red positive area (%) in liver tissues of mice (scale bars, 50 µm, 100µm, 20µm. n=6). (I, J) Expression levels of α-SMA, Col1A1 and Col3A1 in liver tissues of mice were detected by Western blotting and bar plot. All data are presented as the mean ± SD (n =3 independent experiments). Levels of statistical significance are indicated as ***P* < 0.01, ****P* < 0.001, *****P* < 0.0001. One‐way ANOVA with Tukey test analysis and a two‐tailed Student t test were used for statistical analysis.

**
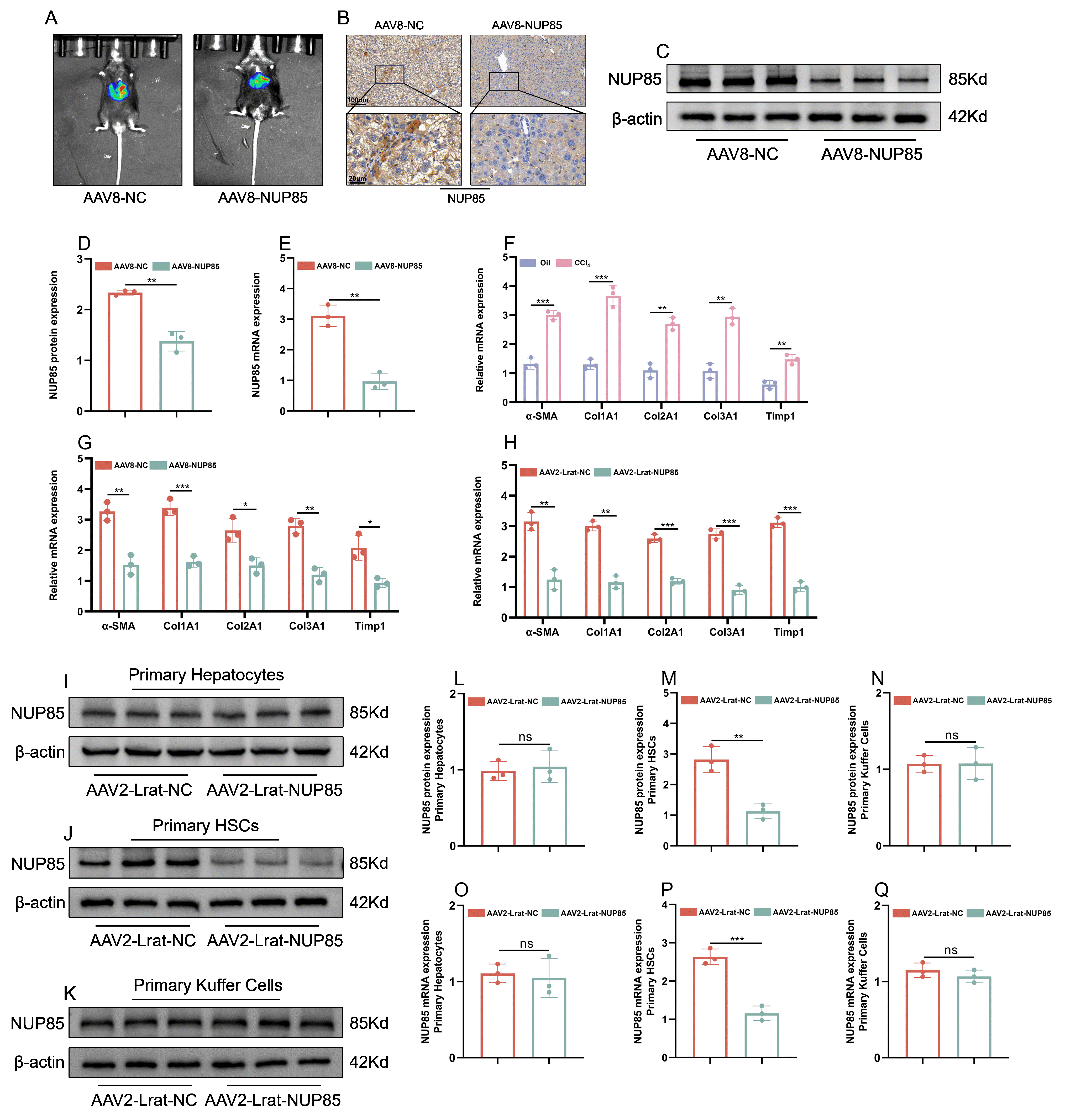
**

**Figure S4.** NUP85 was knockdown in mice liver or HSCs. (A) The distribution of Adeno-Associated Virus was detected by living imaging (n=6). (B) Expression level of NUP85 in liver tissues of mice was detected by IHC (scale bars, 100µm, 20µm. n=6). (C, D) Expression level of NUP85 in liver tissues of mice was detected by Western blotting and bar plot. (E) Expression level of NUP85 in liver tissues of mice was detected by RT-qPCR. (F-H) Expression levels of α-SMA, Col1A1, Col2A1, Col3A1 and Timp1 were detected by RT-qPCR. (I-Q) Expression level of NUP85 was detected by Western blotting and RT-qPCR in primary hepatocytes, primary HSCs, and primary Kuffer cells. All data are presented as the mean ± SD (n =3 independent experiments). Levels of statistical significance are indicated as **P* < 0.05, ***P* < 0.01,****P* < 0.001, “ns” indicates no significance. One‐way ANOVA with Tukey test analysis and a two‐tailed Student t test were used for statistical analysis.


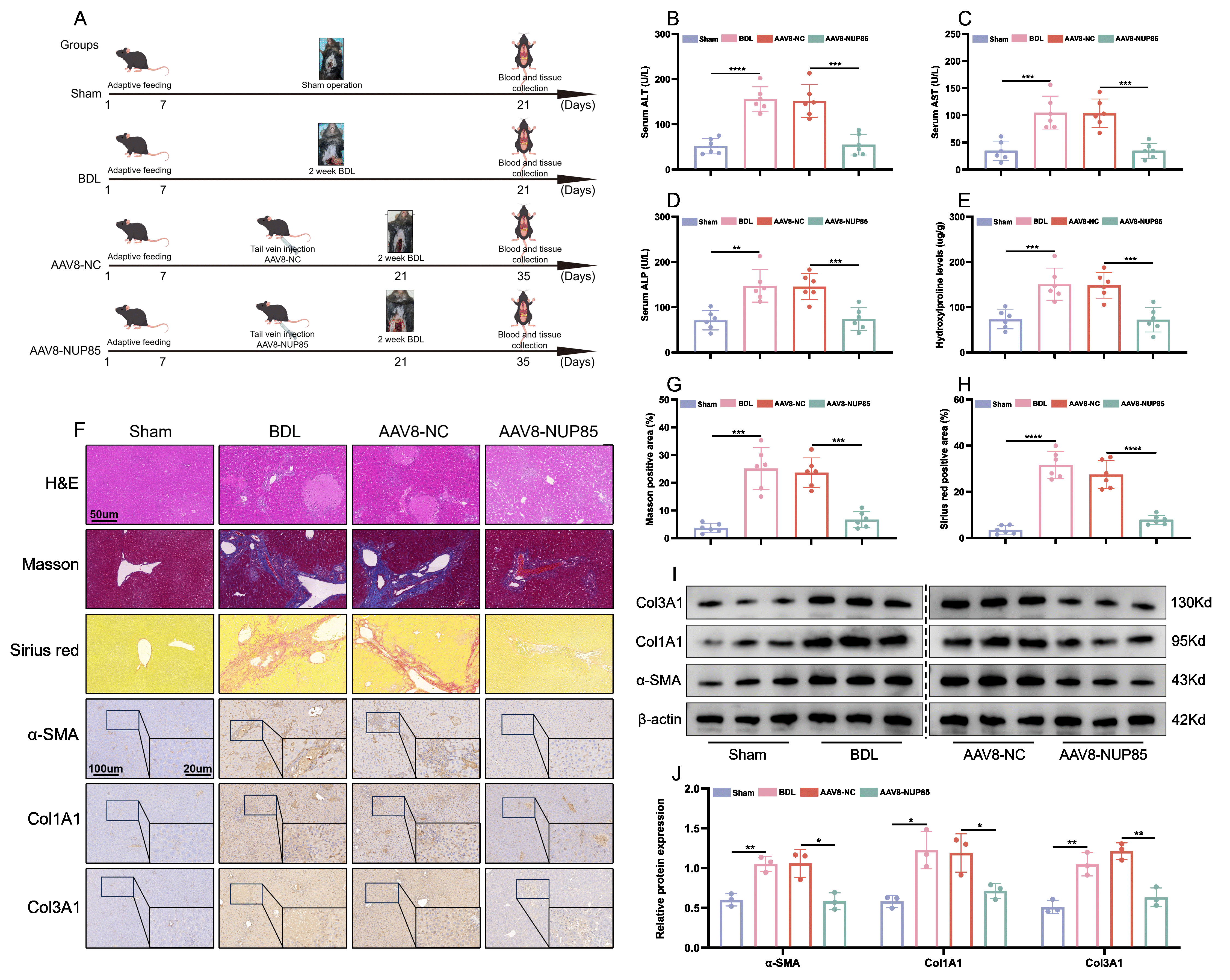


**Figure S5.** Liver-specific NUP85 knockdown alleviates BDL-induced liver fibrosis. (A) Schematic illustrating of the experimental design of AAV8-NUP85 for the treatment of BDL-induced liver fibrosis in mice (n=6). (B-D) Serum expression levels of ALT, AST and ALP in mice (n=6). (E) Liver hydroxyproline content in mice (n=6). (F-H) Representative H&E staining, Masson staining, Sirius red staining, and IHC images, Masson positive area (%) and Sirius red positive area (%) in liver tissues of mice (scale bars, 50 µm, 100µm, 20µm. n=6). (I, J) Expression levels of α-SMA, Col1A1 and Col3A1 in liver tissues of mice were detected by Western blotting and bar plot. All data are presented as the mean ± SD (n =3 independent experiments). Levels of statistical significance are indicated as **P* < 0.05, ***P* < 0.01, ****P* < 0.001, *****P* < 0.0001. One‐way ANOVA with Tukey test analysis and a two‐tailed Student t test were used for statistical analysis.

**
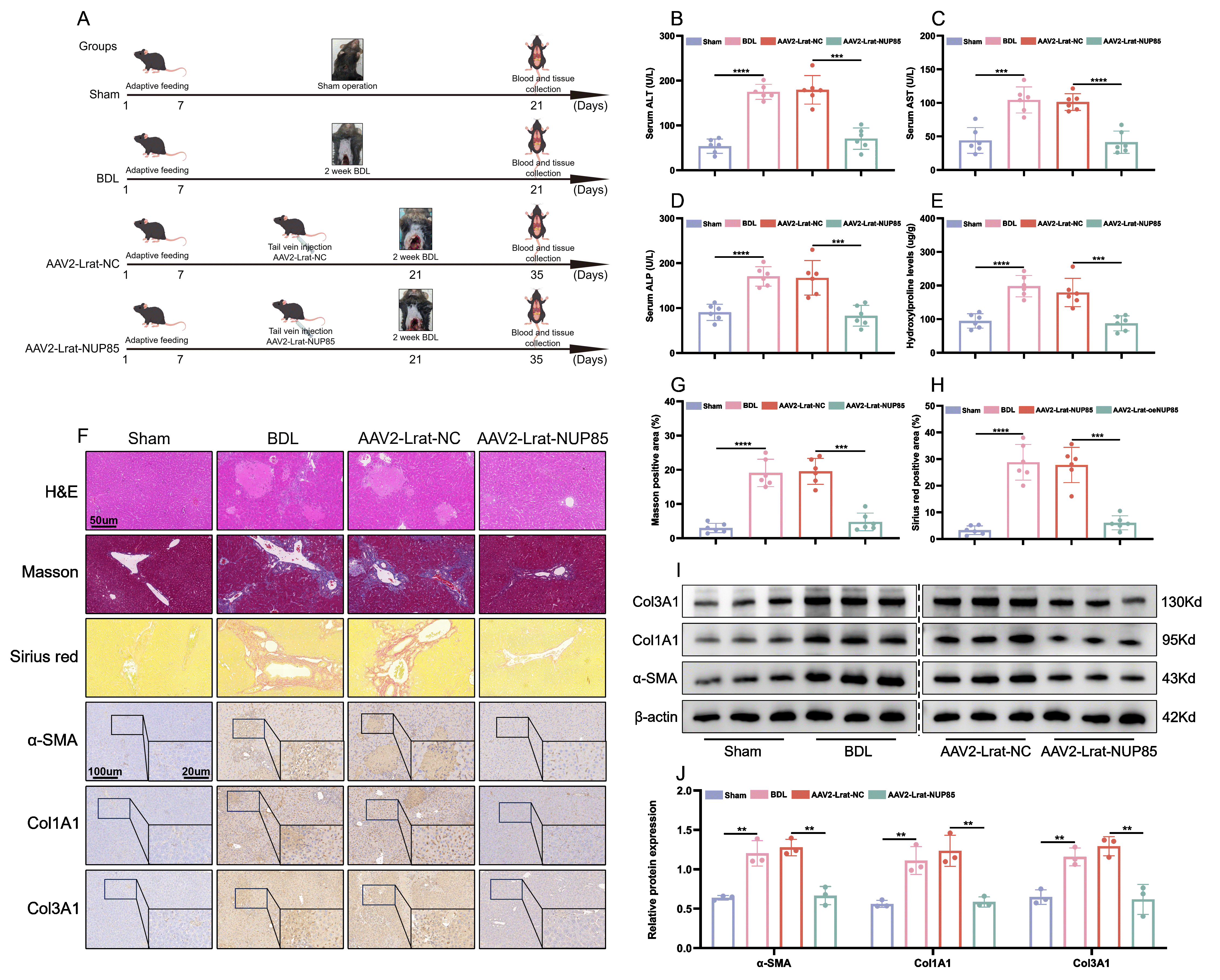
**

**Figure S6.** HSCs-specific NUP85 knockdown alleviates BDL-induced liver fibrosis. (A) Schematic illustrating of the experimental design of AAV2-Lrat-NUP85 for the treatment of BDL-induced liver fibrosis in mice (n=6). (B-D) Serum expression levels of ALT, AST and ALP in mice (n=6). (E) Liver hydroxyproline content in mice (n=6). (F-H) Representative H&E staining, Masson staining, Sirius red staining, and IHC images, Masson positive area (%) and Sirius red positive area (%) in liver tissues of mice (scale bars, 50 µm, 100µm, 20µm n=6). (I, J) Expression levels of α-SMA, Col1A1 and Col3A1 in liver tissues of mice were detected by Western blotting and bar plot. All data are presented as the mean ± SD (n =3 independent experiments). Levels of statistical significance are indicated as ***P* < 0.01, ****P* < 0.001, *****P* < 0.0001. One‐way ANOVA with Tukey test analysis and a two‐tailed Student t test were used for statistical analysis.


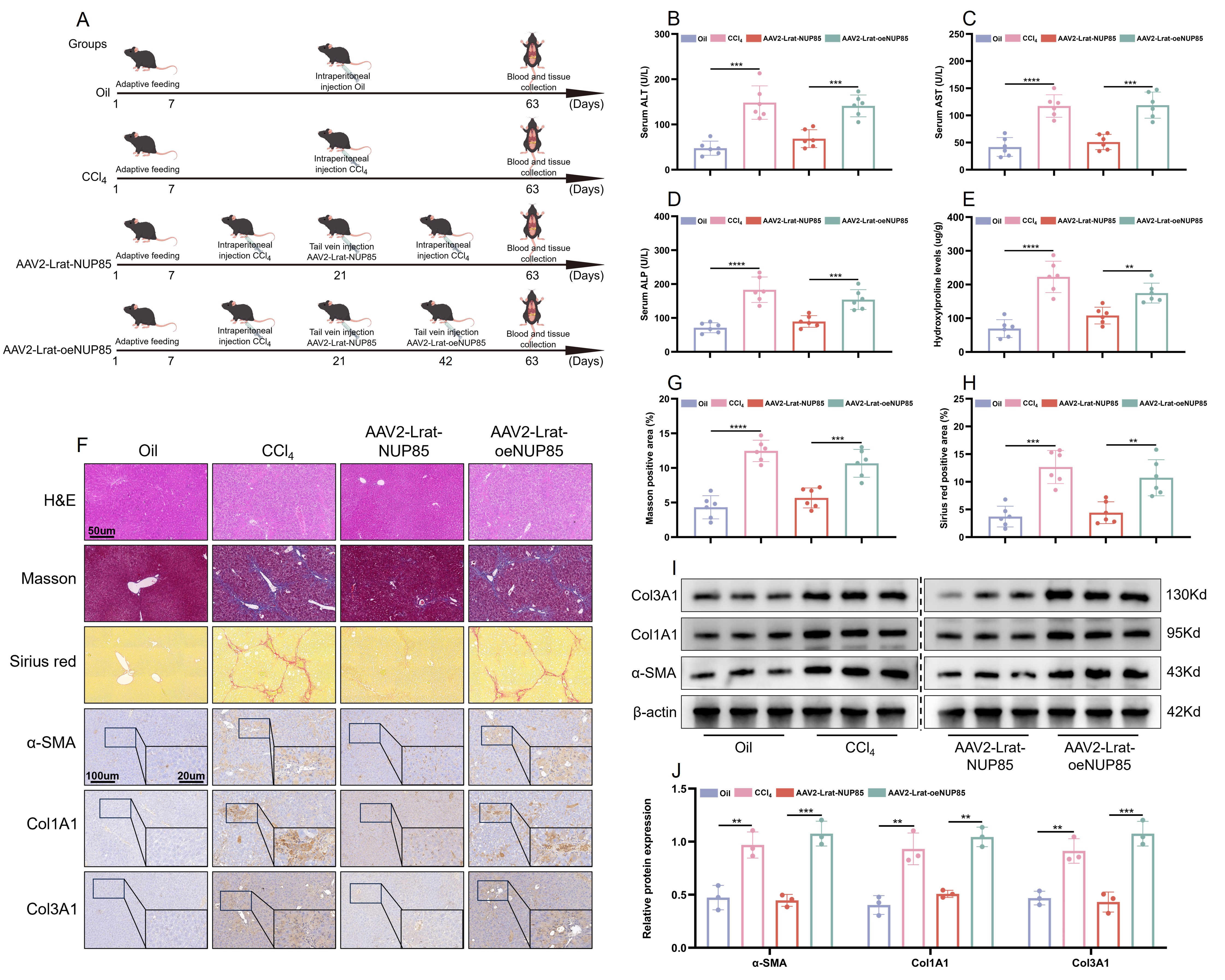


**Figure S7.** HSCs-specific NUP85 overexpression exacerbates CCl_4_-induced liver fibrosis. (A) Schematic illustrating of the experimental design of AAV2-Lrat-NUP85 and AAV2-Lrat-oeNUP85 for the treatment of CCl_4_-induced liver fibrosis in mice (n=6). (B-D) Serum expression levels of ALT, AST and ALP in mice (n=6). (E) Liver hydroxyproline content in mice (n=6). (F-H) Representative H&E staining, Masson staining, Sirius red staining, and IHC images, Masson positive area (%) and Sirius red positive area (%) in liver tissues of mice (scale bars, 50 µm, 100µm, 20µm n=6). (I, J) Expression levels of α-SMA, Col1A1 and Col3A1 in liver tissues of mice were detected by Western blotting and bar plot. All data are presented as the mean ± SD (n =3 independent experiments). Levels of statistical significance are indicated as ***P* < 0.01, ****P* < 0.001, *****P* < 0.0001. One‐way ANOVA with Tukey test analysis and a two‐tailed Student t test were used for statistical analysis.

**
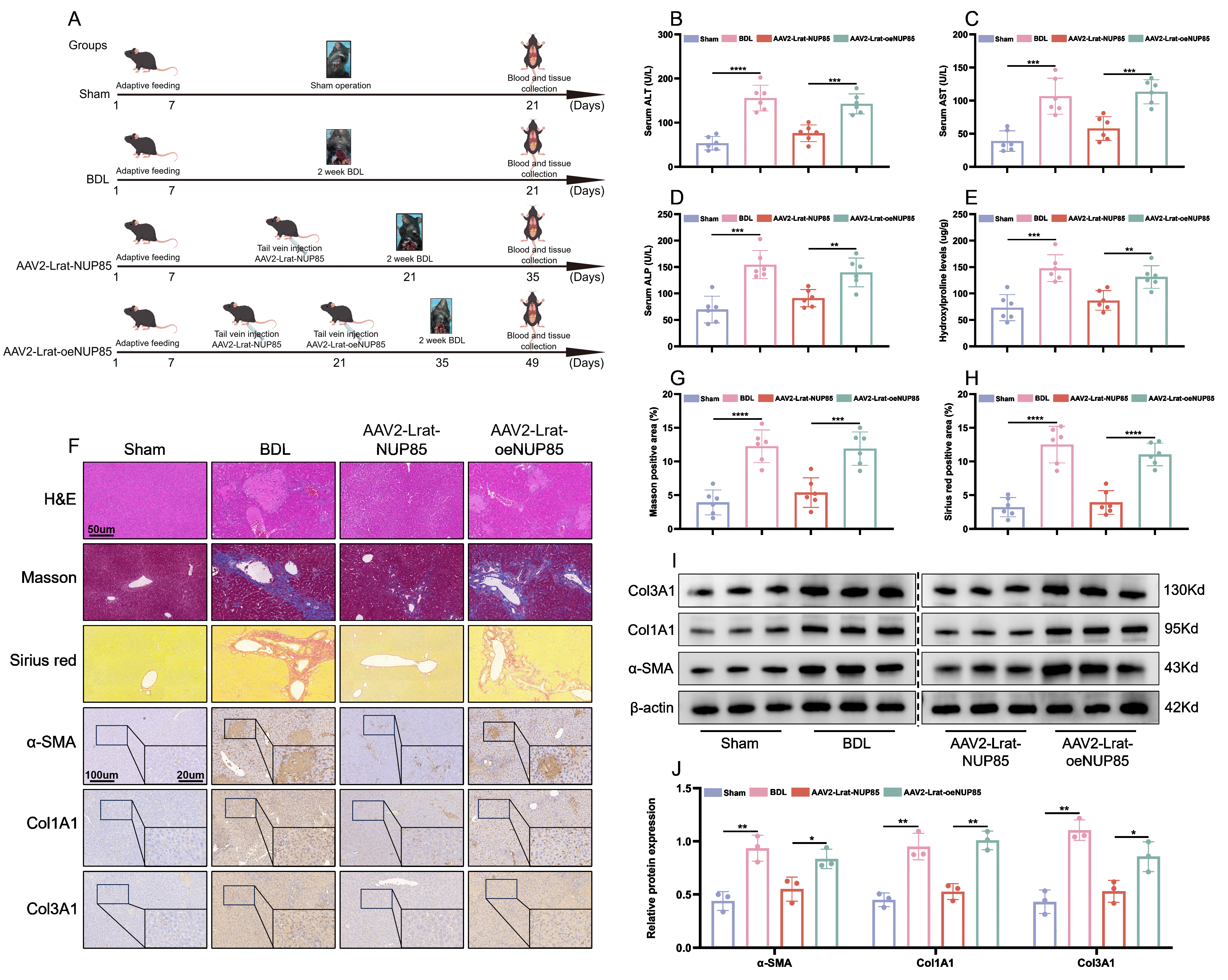
**

**Figure S8.** HSCs-specific NUP85 overexpression exacerbates BDL-induced liver fibrosis. (A) Schematic illustrating of the experimental design of AAV2-Lrat-NUP85 and AAV2-Lrat-oeNUP85 for the treatment of BDL-induced liver fibrosis in mice (n=6). (B-D) Serum expression levels of ALT, AST and ALP in mice (n=6). (E) Liver hydroxyproline content in mice (n=6). (F-H) Representative H&E staining, Masson staining, Sirius red staining, and IHC images, Masson positive area (%) and Sirius red positive area (%) in liver tissues of mice (scale bars, 50 µm, 100µm, 20µm n=6). (I, J) Expression levels of α-SMA, Col1A1 and Col3A1 in liver tissues of mice were detected by Western blotting and bar plot. All data are presented as the mean ± SD (n =3 independent experiments). Levels of statistical significance are indicated as ***P* < 0.01, ****P* < 0.001, *****P* < 0.0001. One‐way ANOVA with Tukey test analysis and a two‐tailed Student t test were used for statistical analysis.

**
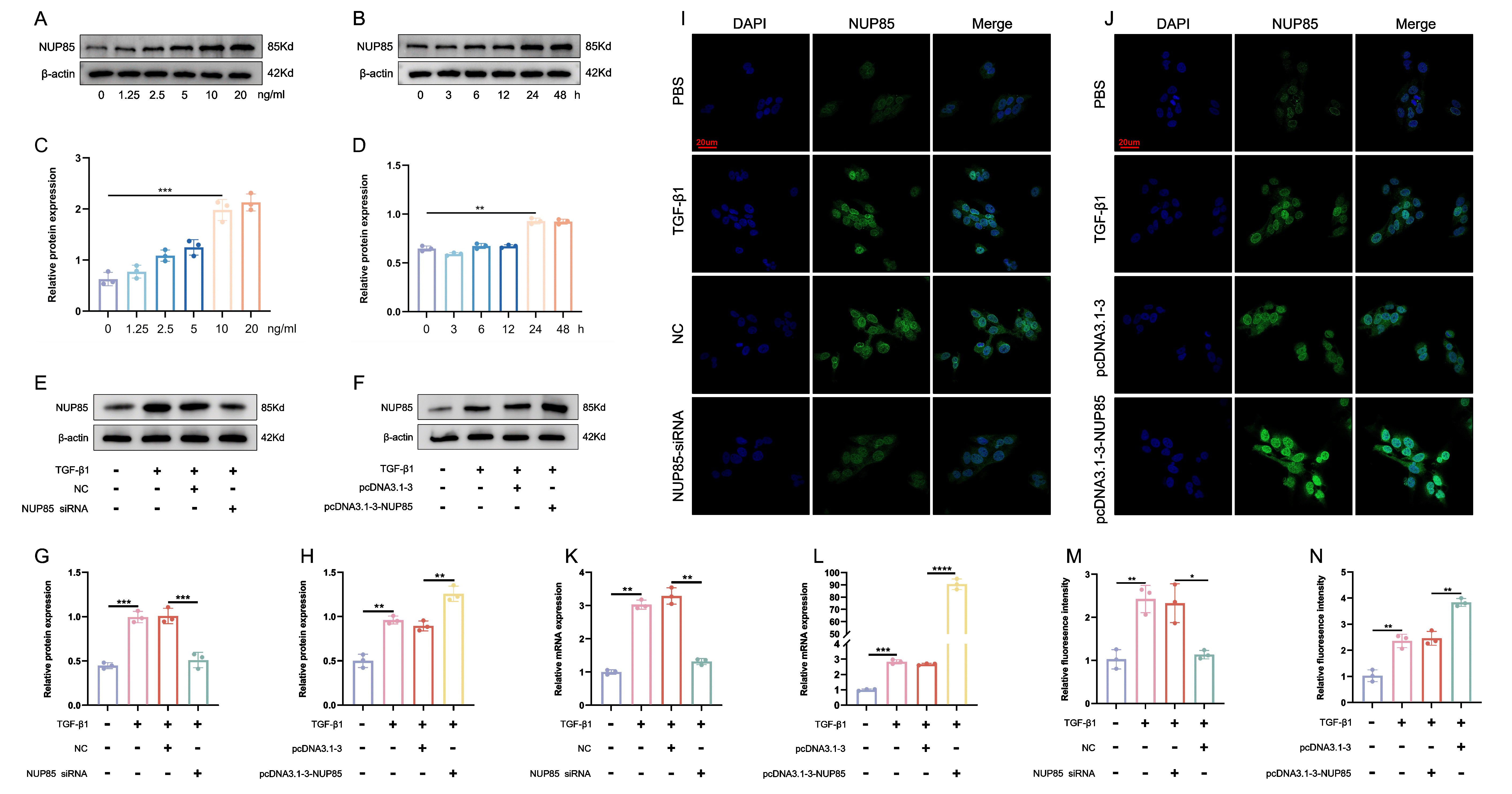
**

**Figure S9.** NUP85 expression level was detected in LX-2 cells. (A-D) Expression level of NUP85 induced by TGF-β1 at different concentrations and times was detected by Western blotting and bar plot in LX-2 cells. (E-H) Expression level of NUP85 was detected by Western blotting and bar plot in LX-2 cells. (I, J, M, N) Expression level of NUP85 was detected by IF and bar plot in LX-2 cells (scale bars, 20 µm). (K, L) Expression level of NUP85 was detected by RT-qPCR in LX-2 cells. All data are presented as the mean ± SD (n =3 independent experiments). Levels of statistical significance are indicated as **P < 0.01, ***P < 0.001, ****P < 0.0001. One‐way ANOVA with Tukey test analysis and a two‐tailed Student t test were used for statistical analysis.

**
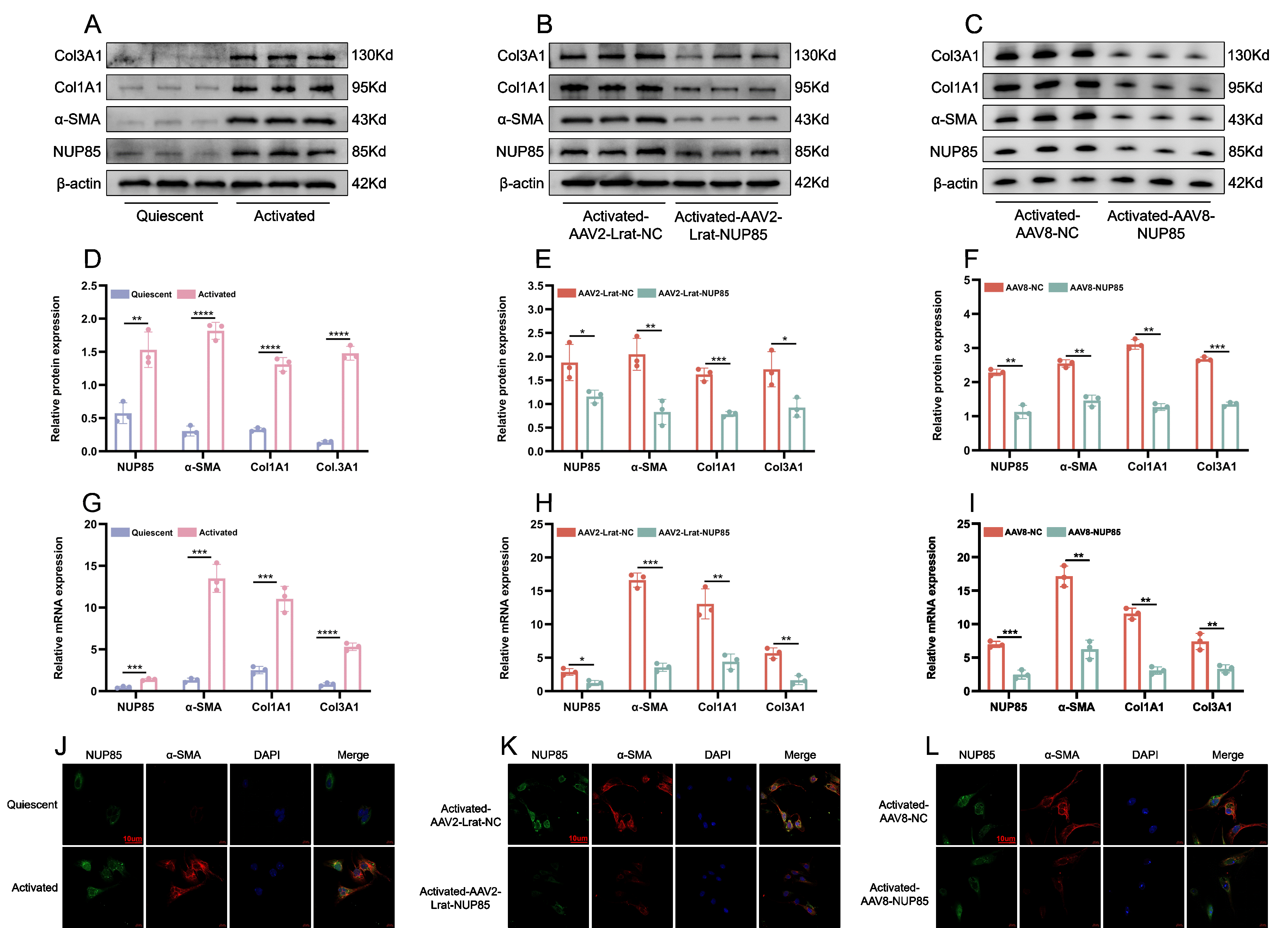
**

**Figure S10.** NUP85 modulates the activation of primary HSCs. (A-F) Expression levels of NUP85, α-SMA, Col1A1 and Col3A1 were detected by Western blotting and bar plot in primary HSCs. (G-I) Expression levels of NUP85, α-SMA, Col1A1 and Col3A1 were detected by RT-qPCR in primary HSCs. (J-L) Expression levels of NUP85, α-SMA, Col1A1 and Col3A1 were detected by IF in primary HSCs (scale bars, 10 µm). All data are presented as the mean ± SD (n =3 independent experiments). Levels of statistical significance are indicated as **P* < 0.05, ***P* < 0.01, ****P* < 0.001, *****P* < 0.0001. One‐way ANOVA with Tukey test analysis and a two‐tailed Student t test were used for statistical analysis.

**
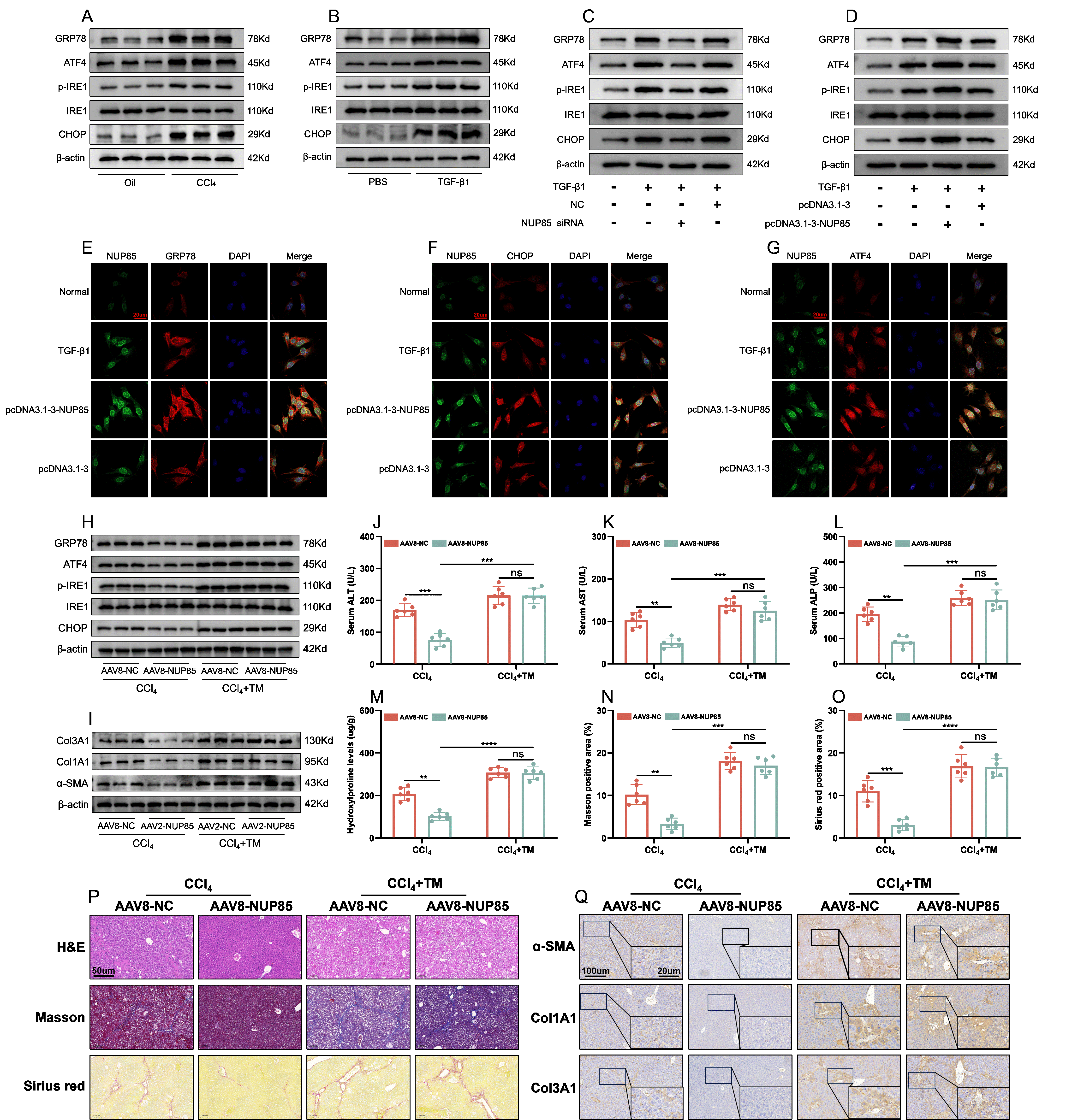
**

**Figure S11.** NUP85 modulates ERS in LX-2 cells. (A-D) Expression levels of GRP78, ATF4, p-IRE1, IPE1 and CHOP were detected by Western blotting in LX-2 cells and CCl_4_-induced fibrosis livers of mice. (E-G) Expression levels of GRP78, ATF4, p-IRE1, IPE1 and CHOP were detected by IF in LX-2 cells and CCl4-induced fibrosis livers of mice (scale bars, 20 µm). (H, I) Expression levels of GRP78, ATF4, p-IRE1, IPE1, CHOP, α-SMA, Col1A1, and Col3A1 in liver tissues of mice were detected by Western blotting. (J-L) Serum expression levels of ALT, AST and ALP in mice (n=6). (M) Liver hydroxyproline content in mice (n=6). (N-Q) Representative H&E staining, Masson staining, Sirius red staining, and IHC images, Masson positive area (%) and Sirius red positive area (%) in liver tissues of mice (scale bars, 50 µm, 100µm, 20µm. n=6). All data are presented as the mean ± SD (n =3 independent experiments). Levels of statistical significance are indicated as ***P* < 0.01, ****P* < 0.001, *****P* < 0.0001, “ns” indicates no significance. One‐way ANOVA with Tukey test analysis and a two‐tailed Student t test were used for statistical analysis.

**
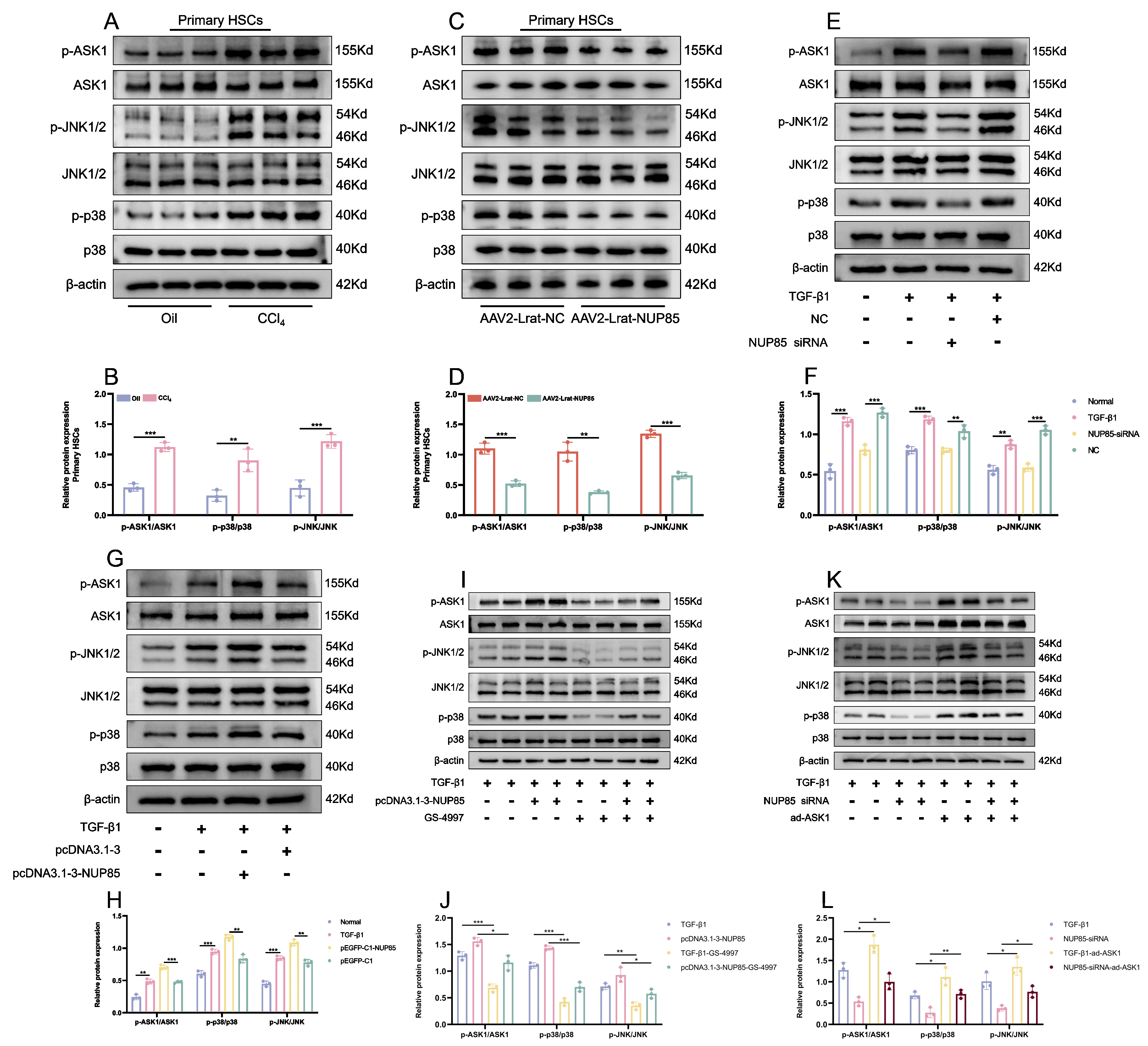
**

**Figure S12.** NUP85 modulates the ASK1-MAPK signaling cascade. (A-D) Expression levels of ASK1, p-ASK1, JNK1/2, p-JNK1/2, p38 and p-p38 were detected by Western blotting and bar plot in Primary HSCs. (E-L) Expression levels of ASK1, p-ASK1, JNK1/2, p-JNK1/2, p38 and p-p38 were detected by Western blotting and bar plot in LX-2 cells. All data are presented as the mean ± SD (n =3 independent experiments). Levels of statistical significance are indicated as **P* < 0.05, ***P* < 0.01, ****P* < 0.001. One‐way ANOVA with Tukey test analysis and a two‐tailed Student t test were used for statistical analysis.

**
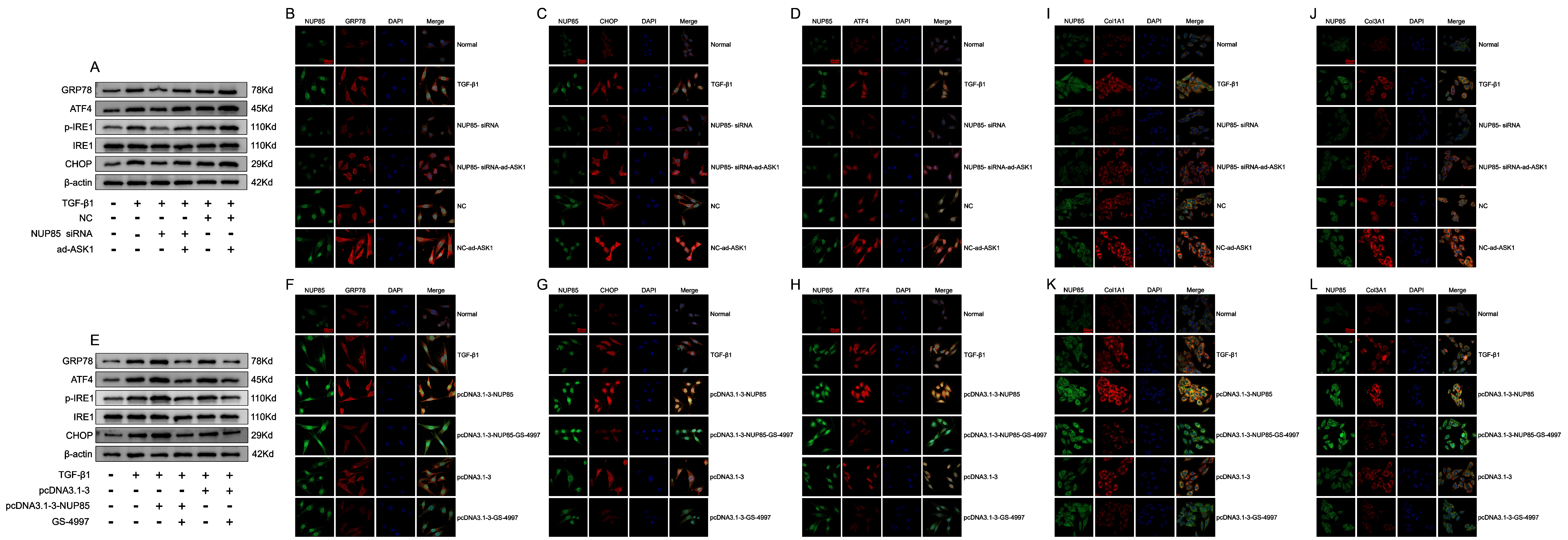
**

**Figure S13.** NUP85 regulates ERS through the ASK1-MAPK signaling cascade. (A, E) Expression levels of GRP78, ATF4, p-IRE1, IPE1 and CHOP were detected by Western blotting in LX-2 cells. (B-D, F-H) Expression levels of GRP78, ATF4, p-IRE1, IPE1 and CHOP were detected by IF in LX-2 cells (scale bars, 20 µm). (I-L) Expression levels of Col1A1 and Col3A1 were detected by IF in LX-2 cells (scale bars, 20 µm). All data are presented as the mean ± SD (n =3 independent experiments). One‐way ANOVA with Tukey test analysis and a two‐tailed Student t test were used for statistical analysis.

**
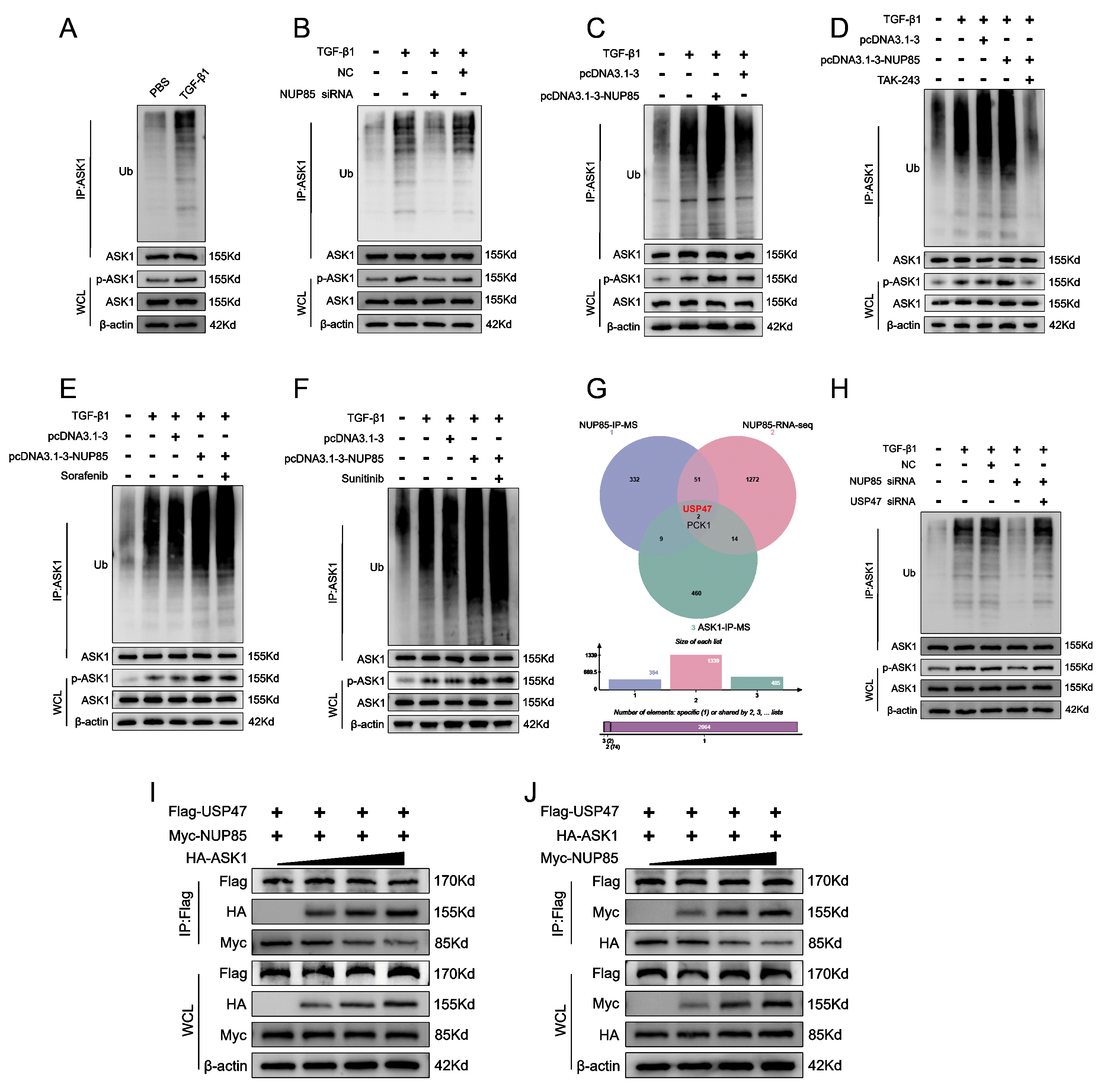
**

**Figure S14.** NUP85 modulates ubiquitination of ASK1 and competes with ASK1 for binding to USP47. (A-F) Ubiquitination level of ASK1 in LX-2 cells was detected by Western blotting. (G) Venn diagram was used to represent the intersection between NUP85-IP-MS, ASK1-IP-MS, and NUP85-RNA-seq. (H) Ubiquitination level of ASK1 in LX-2 cells was detected by Western blotting. (I) CO-IP was used to detect the effect of ASK1 on NUP85 binding to USP47. (J) CO-IP was used to detect the effect of NUP85 on ASK1 binding to USP47. All data are presented as the mean ± SD (n =3 independent experiments).

**
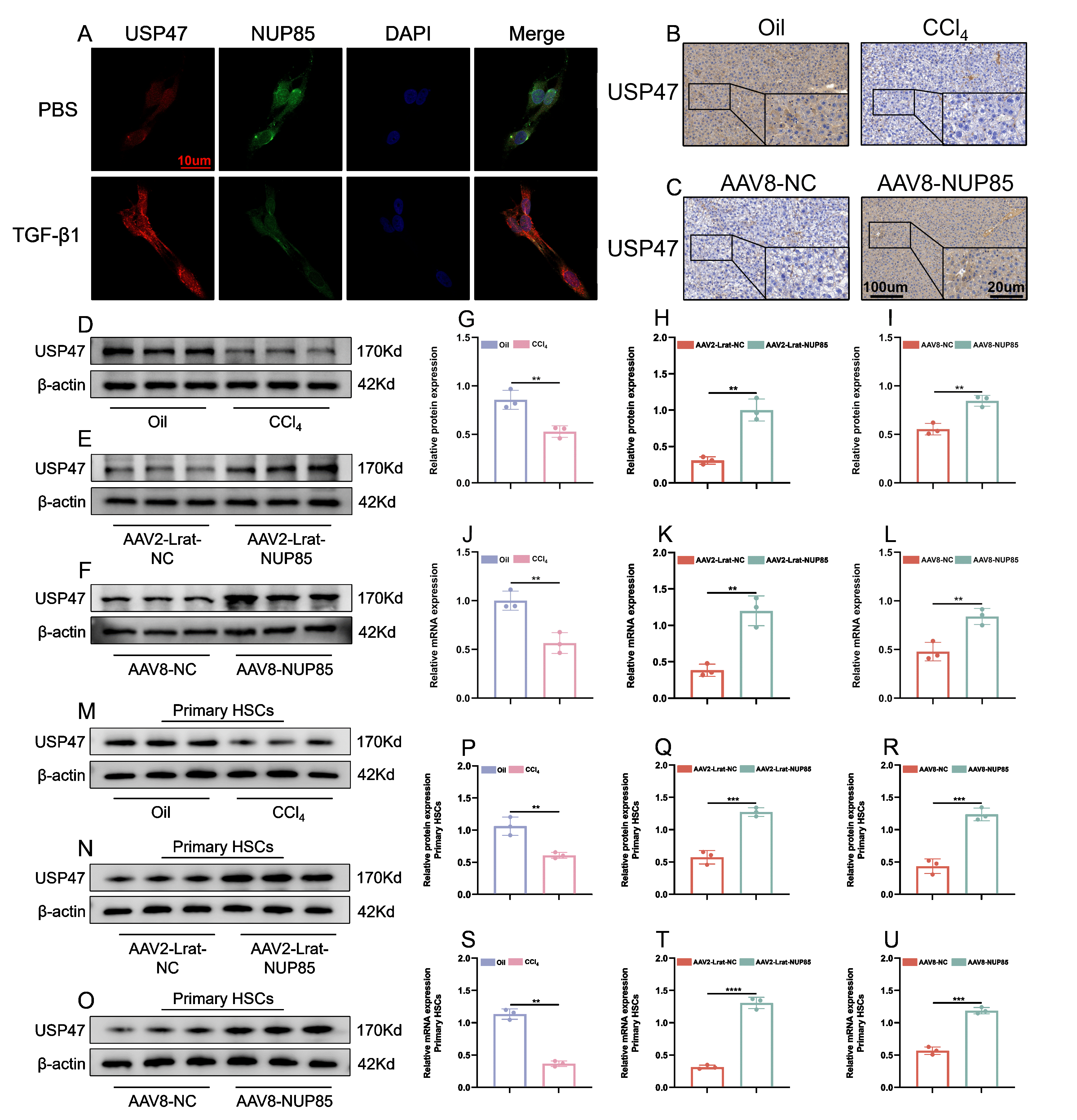
**

**Figure S15.** The expression level of USP47 was regulated by NUP85. (A) Expression levels of NUP85 and USP47 were detected by IF in LX-2 cells (scale bars, 10 µm). (B, C) Expression level of USP47 in liver tissues of mice was detected by IHC (scale bars, 100µm, 20 µm. n=6). (D-I) Expression level of USP47 in liver tissues of mice was detected by Western blotting and bar plot. (J-L) Expression level of USP47 in liver tissues of mice was detected by RT-qPCR. (M-R) Expression level of USP47 in primary HSCs was detected by Western blotting and bar plot. (S-U) Expression level of USP47 in primary HSCs was detected by RT-qPCR. All data are presented as the mean ± SD (n =3 independent experiments). Levels of statistical significance are indicated as ***P* < 0.01, ****P* < 0.001, *****P* < 0.0001. One‐way ANOVA with Tukey test analysis and a two‐tailed Student t test were used for statistical analysis.

**
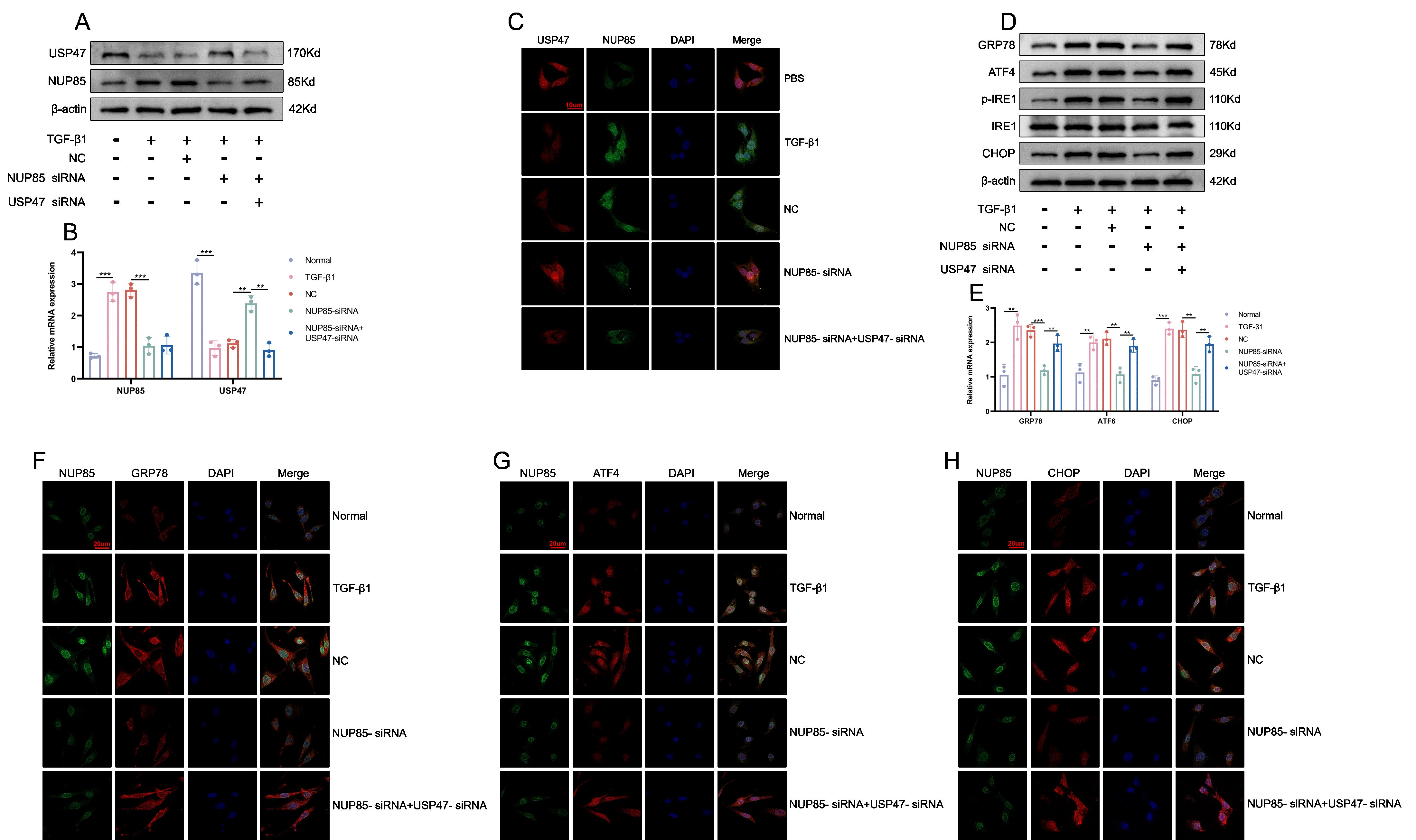
**

**Figure S16.** NUP85 regulates ERS through USP47. (A-C) Expression levels of NUP85 and USP47 were detected by Western blotting, RT-qPCR, and IF in LX-2 cells (scale bars, 20 µm). (D, E) Expression levels of GRP78, ATF4, p-IRE1, IPE1 and CHOP were detected by Western blotting, and RT-qPCR in LX-2 cells. (F-H) Expression levels of GRP78, ATF4, and CHOP were detected by IF in LX-2 cells (scale bars, 20 µm). All data are presented as the mean ± SD (n =3 independent experiments). Levels of statistical significance are indicated as ***P* < 0.01, ****P* < 0.001. One‐way ANOVA with Tukey test analysis and a two‐tailed Student t test were used for statistical analysis.

**
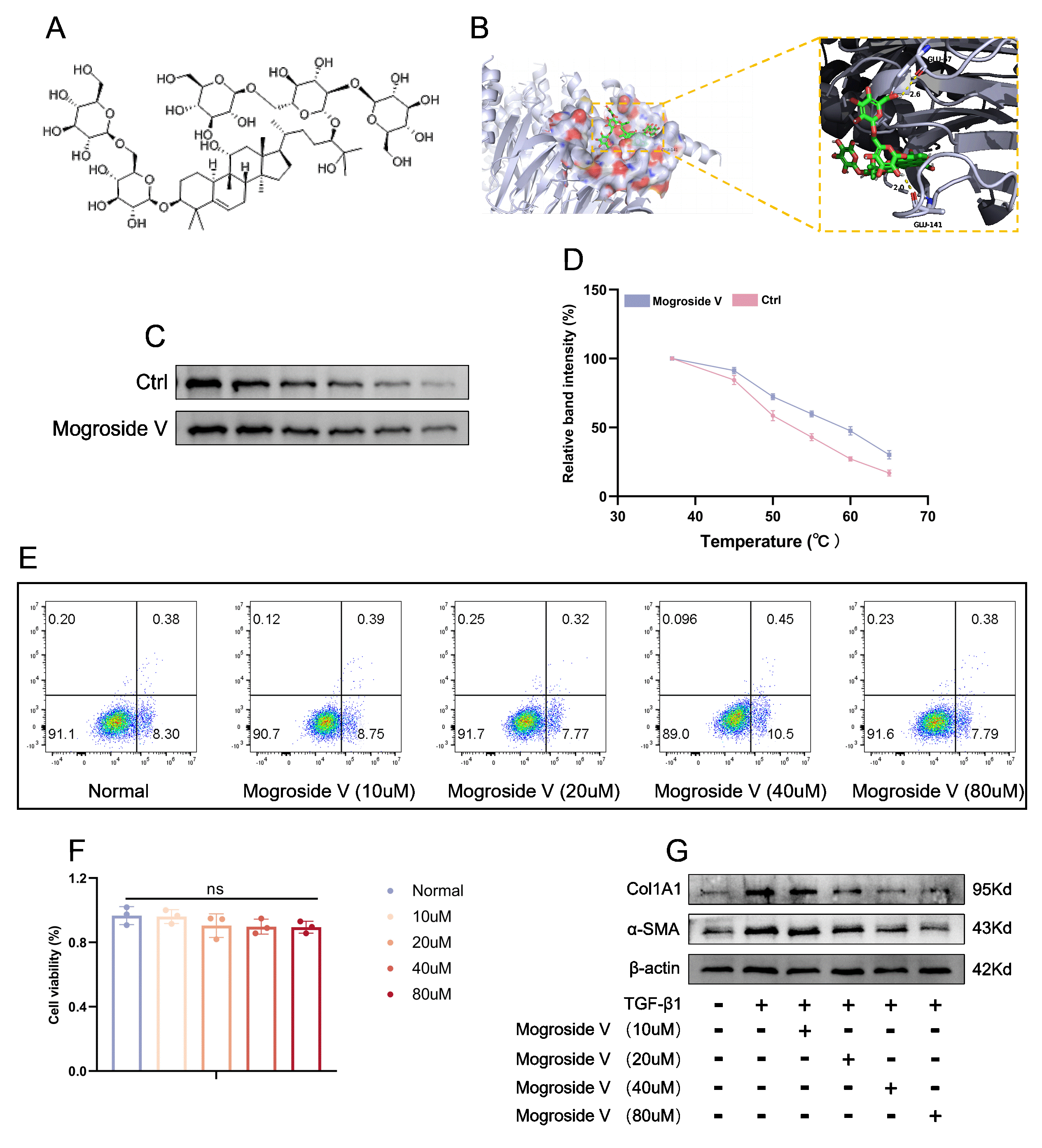
**

**Figure S17.** Mogroside V inhibits NUP85 to alleviate liver fibrosis. (A) Schematic structure of Mogroside V. (B) Binding site of Mogroside V to NUP85. (C, D) LX-2 cells were cultivated with or without Mogroside V (80 um) for 24 h and CETSA test was performed. (E) Flow cytometry was used to detect the viability of LX-2 cells. (F) CCK-8 was used to detect the viability of LX-2 cells. (G) Expression levels of α-SMA and Col1A1 in LX-2 cells were detected by Western blotting. All data are presented as the mean ± SD (n =3 independent experiments). Levels of statistical significance are indicated as “ns” indicates no significance. One‐way ANOVA with Tukey test analysis and a two‐tailed Student t test were used for statistical analysis.

**
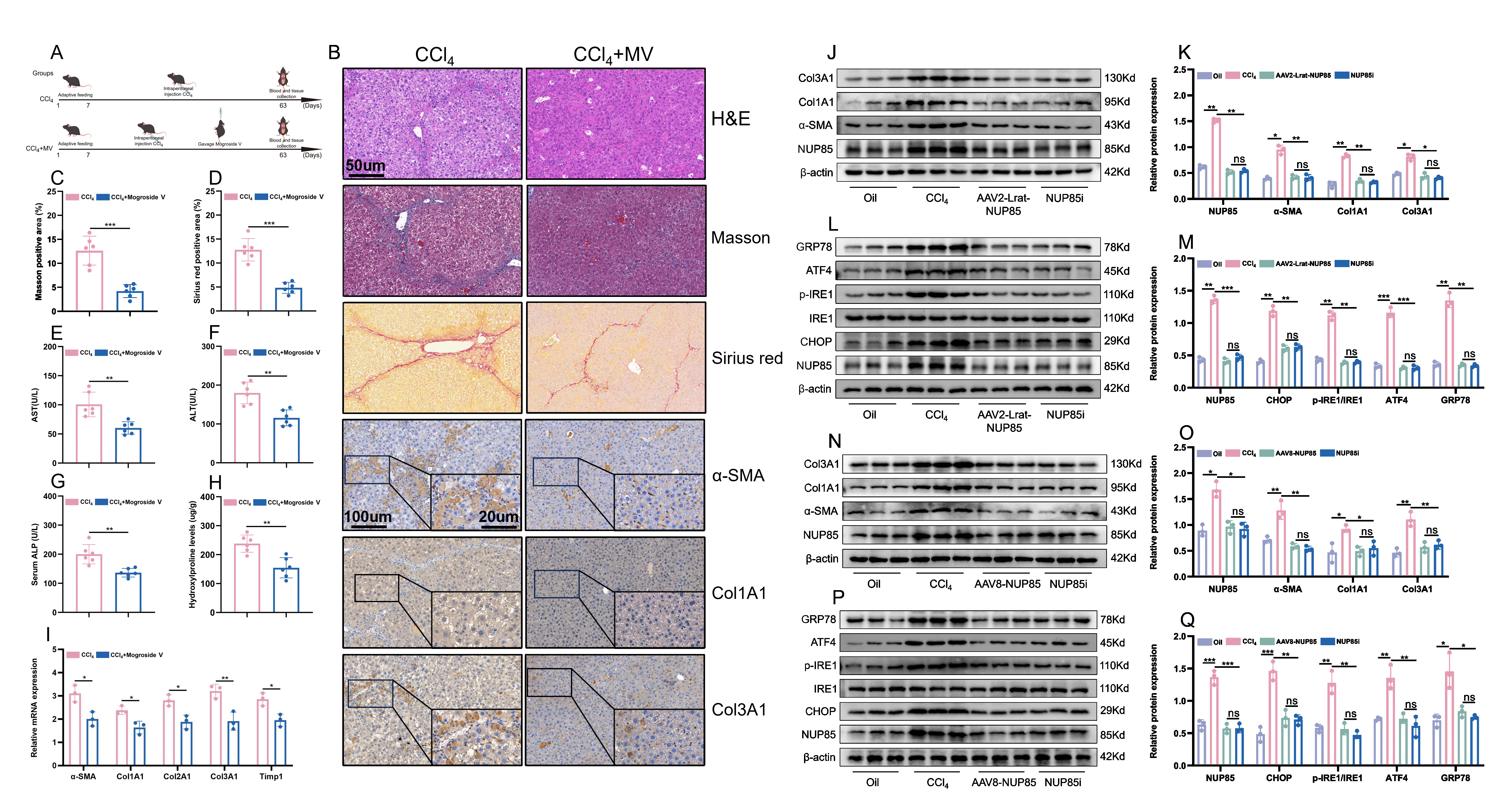
**

**Figure S18.** MV alleviates CCl_4_-induced liver fibrosis. (A) Schematic diagram of mice treatment (n=6). (B-D) Representative H&E staining, Masson staining, Sirius red staining and IHC images, Masson positive area (%) and Sirius red positive area (%) in liver tissues of mice (scale bars, 50 µm, 100µm, 20µm. n=6). (E-G) Serum expression levels of ALT, AST and ALP (n=6). (H) Liver hydroxyproline content (n=6). (I-Q) Expression levels of NUP85, α-SMA, Col1A1, Col3A1, GRP78, ATF4, p-IRE1, and CHOP were detected by Western blotting and RT-qPCR. All data are presented as the mean ± SD (n =3 independent experiments). Levels of statistical significance are indicated as **P* < 0.05, ***P* < 0.01; ****P* < 0.001, “ns” indicates no significance. One‐way ANOVA with Tukey test analysis and a two‐tailed Student t test were used for statistical analysis.

**
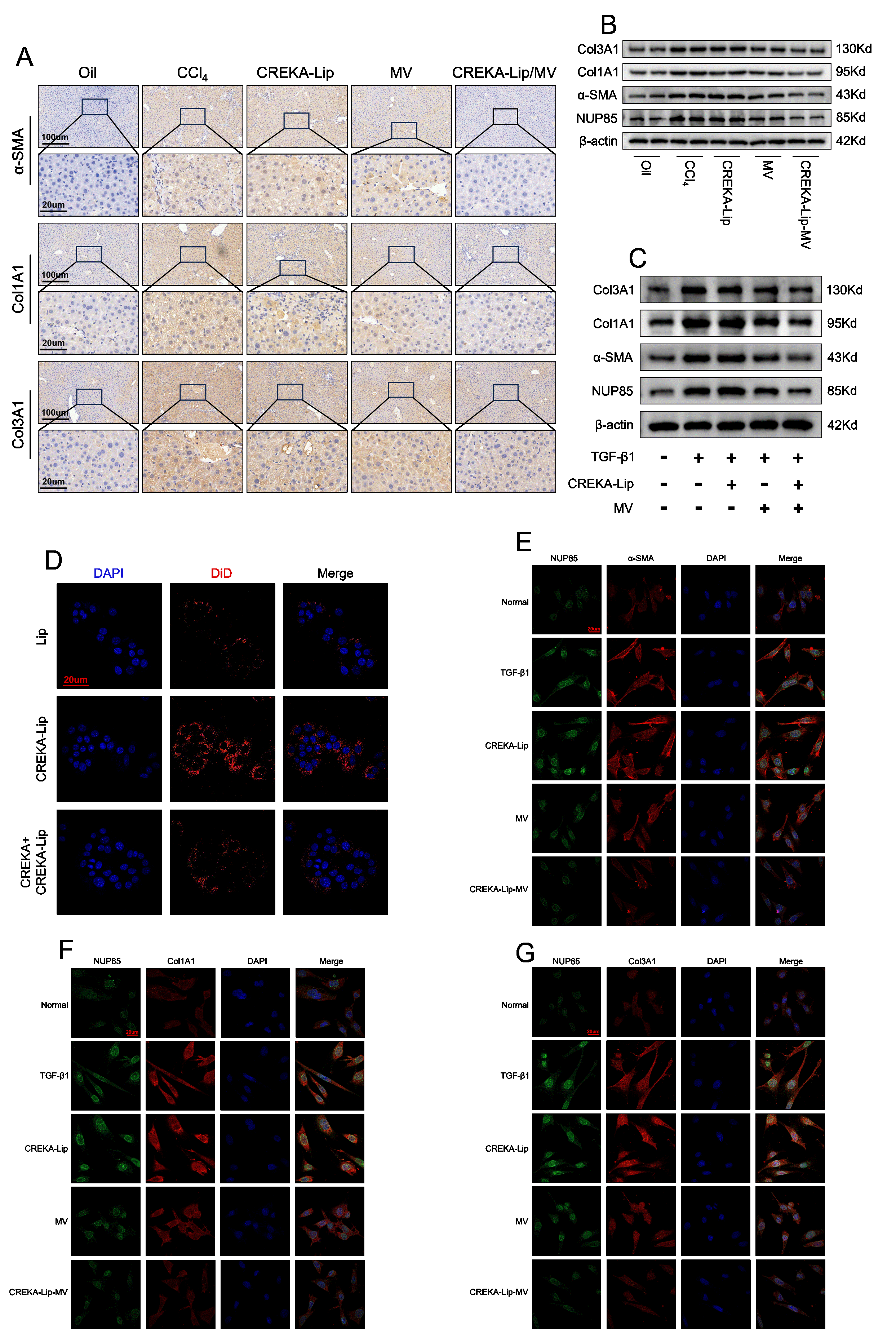
**

**Figure S19.** CREKA-Lip/MV effectively attenuates liver fibrosis. (A) Representative IHC images in liver tissues of mice (scale bars, 100µm, 20µm. n=6). (B, C) Expression levels of NUP85, α-SMA, Col1A1 and Col3A1 were detected by Western blotting. (D) Representative confocal microscopy images showing the cellular uptake of liposomes in LX-2 cells (scale bars, 20 µm). (E-G) Expression levels of α-SMA, Col1A1 and Col3A1 were detected by IF in LX-2 cells (scale bars, 20 µm). All data are presented as the mean ± SD (n =3 independent experiments). One‐way ANOVA with Tukey test analysis and a two‐tailed Student t test were used for statistical analysis.

**
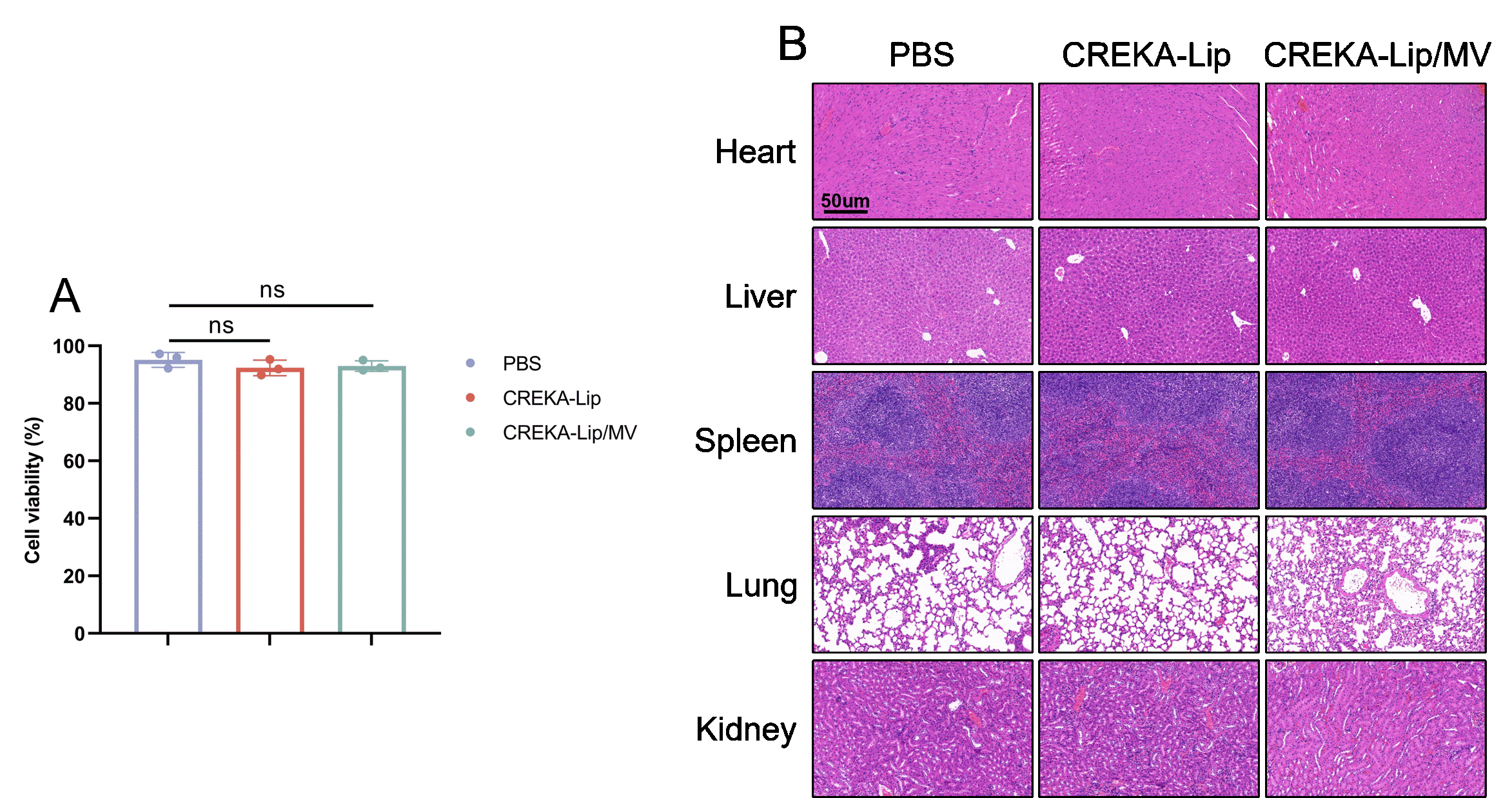
**

**Figure S20.** MV has good biosafety. (A) CCK-8 was used to detect the viability of LX-2 cells. (B) Representative H&E staining images in major organs of mice (scale bars, 50µm. n=6). All data are presented as the mean ± SD (n =3 independent experiments). Levels of statistical significance are indicated as “ns” indicates no significance. One‐way ANOVA with Tukey test analysis and a two‐tailed Student t test were used for statistical analysis.

| **Antibody** | **Manufacturer** | **Catalog Number** | **Source** | **Dilutions** |
| --- | --- | --- | --- | --- |
| β-actin | Abcam | ab8226 | Mouse | 1:2000 |
| NUP85 | Santa Cruz | sc-376111 | Mouse | 1:1000 |
| α-SMA | Proteintech | 14395-1-AP | Rabbit | 1:1000 |
| Col1A1 | Abcam | ab270993 | Rabbit | 1:1000 |
| Col3A1 | Abcam | ab184993 | Rabbit | 1:1000 |
| CHOP | Proteintech | 15204-1-AP | Rabbit | 1:1000 |
| IRE1 | Abcam | ab37073 | Rabbit | 1:1000 |
| p-IRE1 | Abcam | ab48187 | Rabbit | 1:1000 |
| ATF4 | Proteintech | 10835-1-AP | Rabbit | 1:1000 |
| GRP78 | Proteintech | 11587-1-AP | Rabbit | 1:1000 |
| p38 | Proteintech | 14064-1-AP | Rabbit | 1:1000 |
| p-p38 | Proteintech | 28796-1-AP | Rabbit | 1:1000 |
| JNK1/2 | Thermo Fisher | 279Q38 | Rabbit | 1:1000 |
| p-JNK1/2 | Thermo Fisher | D12H7L17 | Rabbit | 1:1000 |
| ASK1 | Abcam | ab45178 | Rabbit | 1:1000 |
| p-ASK1 | Abcam | ab278547 | Rabbit | 1:1000 |
| USP47 | Abcam | ab72143 | Rabbit | 1:1000 |
| Ub | Santa Cruz | sc-53509 | Mouse | 1:1000 |

**Table S1.** Antibodies used in Western blotting.

| **RT-qPCR Primers** | **Forward primer** | **Reverse primer** |
| --- | --- | --- |
| α-SMA (Human) | AAAAGACAGCTACGTGGGTGA | GCCATGTTCTATCGGGTACTTC |
| Col1A1 (Human) | GTGCGATGACGTGATCTGTGA | CGGTGGTTTCTTGGTCGGT |
| Col3A1 (Human) | GGAGCTGGCTACTTCTCGC | GGGAACATCCTCCTTCAACAG |
| α-SMA (Mouse) | CCCAGACATCAGGGAGTAATGG | TCTATCGGATACTTCAGCGTCA |
| Col1A1 (Mouse) | GCTCCTCTTAGGGGCCACT | ATTGGGGACCCTTAGGCCAT |
| Col2A1 (Mouse) | GGGTCACAGAGGTTACCCAG | ACCAGGGGAACCACTCTCAC |
| Col3A1 (Mouse) | CTGTAACATGGAAACTGGGGAAA | CCATAGCTGAACTGAAAACCACC |
| Timp1 (Mouse) | CGAGACCACCTTATACCAGCG | ATGACTGGGGTGTAGGCGTA |
| NUP85 (Human) | GGCGAGCCAACAGTCACTTT | ACTCTTCGTCAATTCTCTGGAGG |
| NUP85 (Mouse) | GAGCCCGCAGTAACTTGGATT | TGCCTGTTTGGTTGAAGGAAG |
| USP47 (Human) | CTCGACGCTAATTTTGAGCCA | CTCTTGGAAGCGGACCTATAAAC |
| USP47 (Mouse) | GATGTGATTCCCTTGGATTGCT | AACCCCATTGGTGTATCTTCTTC |
| GRP78 (Human) | CATCACGCCGTCCTATGTCG | CGTCAAAGACCGTGTTCTCG |
| ATF4 (Human) | ATGACCGAAATGAGCTTCCTG | GCTGGAGAACCCATGAGGT |
| CHOP (Human) | GGAAACAGAGTGGTCATTCCC | CTGCTTGAGCCGTTCATTCTC |

**Table S2.** Primer sequence used in RT-qPCR.

| **Antibody** | **Manufacturer** | **Catalog Number** | **Source** | **Dilutions** |
| --- | --- | --- | --- | --- |
| NUP85 | Proteintech | 83288-2-RR | Rabbit | 1:100 |
| NUP85 | Santa Cruz | sc-376111 | Mouse | 1:100 |
| α-SMA | Proteintech | 14395-1-AP | Rabbit | 1:100 |
| Col1A1 | Abcam | ab270993 | Rabbit | 1:100 |
| Col3A1 | Abcam | ab184993 | Rabbit | 1:100 |
| CHOP | Proteintech | 15204-1-AP | Rabbit | 1:100 |
| ATF4 | Proteintech | 10835-1-AP | Rabbit | 1:100 |
| GRP78 | Proteintech | 11587-1-AP | Rabbit | 1:100 |
| ASK1 | Abcam | ab45178 | Rabbit | 1:100 |
| USP47 | Santa Cruz | sc-100633 | Mouse | 1:100 |

**Table S3.** Antibodies used in IF.

| **Antibody** | **Manufacturer** | **Catalog Number** | **Source** | **Dilutions** |
| --- | --- | --- | --- | --- |
| NUP85 | Santa Cruz | sc-376111 | Mouse | 1:100 |
| α-SMA | Proteintech | 14395-1-AP | Rabbit | 1:100 |
| Col1A1 | Abcam | ab270993 | Rabbit | 1:100 |
| Col3A1 | Abcam | ab184993 | Rabbit | 1:100 |
| USP47 | Abcam | ab72143 | Rabbit | 1:100 |

**Table S4.** Antibodies used in IHC.
